# Do Humans Use Push‐Down Stacks When Learning or Producing Center‐Embedded Sequences?

**DOI:** 10.1111/cogs.70112

**Published:** 2025-09-15

**Authors:** Stephen Ferrigno, Samuel J. Cheyette, Susan Carey

**Affiliations:** ^1^ Department of Psychology University of Wisconsin‐Madison; ^2^ Department of Psychology Harvard University; ^3^ Department of Brain and Cognitive Sciences Massachusetts Institute of Technology

**Keywords:** Recursion, Center‐embedded structures, Cross‐serial structures, Artificial grammar, Sequence learning

## Abstract

Complex sequences are ubiquitous in human mental life, structuring representations within many different cognitive domains—natural language, music, mathematics, and logic, to name a few. However, the representational and computational machinery used to learn abstract grammars and process complex sequences is unknown. Here, we used an artificial grammar learning task to study how adults abstract center‐embedded and cross‐serial grammars that generalize beyond the level of embedding of the training sequences. We tested untrained generalizations to longer sequence lengths and used error patterns, item‐to‐item response times, and a Bayesian mixture model to test two possible memory architectures that might underlie the sequence representations of each grammar: stacks and queues. We find that adults learned both grammars, that the cross‐serial grammar was easier to learn and produce than the matched center‐embedded grammar, and that item‐to‐item touch times during sequence generation differed systematically between the two types of sequences. Contrary to widely held assumptions, we find no evidence that a stack architecture is used to generate center‐embedded sequences in an indexed A^n^B^n^ artificial grammar. Instead, the data and modeling converged on the conclusion that both center‐embedded and cross‐serial sequences are generated using a queue memory architecture. In this study, participants stored items in a first‐in‐first‐out memory architecture and then accessed them via an iterative search over the stored list to generate the matched base pairs of center‐embedded or cross‐serial sequences.

All animals capable of goal‐directed activity have the capacity to represent specific ordered sequences. This capacity is required for action planning, representing song (as in birdsong), and many activities that animals, at least as complex as fish and insects, engage in. Sets of specific sequences can also share abstract structure (think of arias in Handel's operas, A B A, or simple sentences with transitive verbs such as “she hit the ball, Steve kissed his wife…”). Humans, at least, learn and execute generative procedures, that is, learn and execute grammars, for creating novel sequences that share an abstract structure. An example of an abstract structure shared across sequences is center‐embedding, where elements of a specific sequence are contained within other elements of the same sequence. Center embedding is seen in natural language (e.g., the dog the cat chased ran away) but can also be seen across many other domains. In mathematics and computer programming, the structure of nested parentheses follows an abstract, but common center‐embedding structure, such that the innermost parenthetical needs to be closed off before the outer ones (a common source of bugs in code). In action planning, there are many complex sequences of that have this same center‐embedded structure such as stacking Russian nesting dolls, making a sandwich (e.g., fillings go on the inside of the bread elements, and a jar of peanut butter needs to be opened before scooping it out and then closed after scooping), and generating plans for games like the tower of Hanoi or solving a Rubik's Cube.

Herb Terrace carried out some of the first systematic studies of nonlinguistic animals’ capacity to learn *specific* ordered lists (2005). Terrace showed that rhesus macaques can learn to sequence arbitrary lists up to at least 8 items (e.g., the 4‐item list: touch the star, then the bottle, then the square, then the bell). In these studies, the items to be ordered are presented in random spatial arrays that change on each trial. The order of the list must be mentally represented for the animal to touch the items in the correct order. These studies found that monkeys and humans can use two different associative mechanisms to represent specific arbitrary lists—1: associative chaining and 2: ordinal position learning (see Ferrigno, 2022, for a review of the evidence for *both*). Associative chaining allows an animal to learn a sequence by building a chain of rewarded stimulus‐response units. For example, an animal trained on the above list would build up associative mappings between A(star) –> B(bottle) and B(bottle) –> C(square) and C(square) –> D(bell). This type of pairwise associative chaining has been found to underlie the acquisition of songs in some bird species, such as finches (Lipkind et al., 2013). Second, animals, including humans, can represent specific sequences by associating specific items with representations of ordinal position (e.g., position 1: star, position 2: bottle, position 3: square, and position 4: bell; Terrace, 2005).

Here, we use the methodology Terrace developed to study the memory structures deployed in the production of specific sequences that share abstract structures. The memory structures must abstract away from any specific sequence; that is, they cannot be the associative mechanisms described above. For example, “1 2 3 3 2 1,” “a b c c b a,” and “({[]})” are all center‐embedded sequences. The associative mechanisms Terrace (2005) and others documented cannot represent even simple abstract grammars; these associative mechanisms are item‐specific. Only the position of a specific item (ordinal position) or the item following a specific item (associative chaining) is represented. Thus, neither associative chaining nor ordinal position learning can be used to generate all the sequences licensed by abstract grammars.

In the 1950s, linguists and computer scientists (e.g., Chomsky, 1956; Chomsky & Schützenberger, 1959) began a project of characterizing the grammars that can generate all and only the sequences constructable from a fixed set of primitive atomic individuals. They distinguished different classes of grammars, that is, they distinguished different sequence‐generating devices. We follow the terminology they introduced in characterizing sequence structures at various levels of abstraction:

*Primitive Vocabulary*—The set of atomic symbols from which the sequences in a language are composed.

*Sequence*—A specific sequence of symbols drawn from the primitive vocabulary.

*Grammar*—Abstract characterization or generative device that determines all and only the sequences in a language.

*Language*—The set of sequences that satisfy a grammar.

This terminology applies to natural language grammars, artificial grammars, and any grammars that are a set of sequences that share an abstract structure. Chomsky's and Schützenberger's initial work distinguished four types of grammars that differed in complexity: regular grammars, deploying finite state automata; context‐free grammars, deploying push‐down automata; context‐sensitive grammars, deploying linear bounded automata; and unrestricted grammars, deploying a Turing machine. They showed that these grammars are distinguished by the working memory demands of representing earlier encountered parts of a given sequence in determining a full sequence that is licensed by the grammar.

*Finite state grammars* require no working memory of the specific sequence at the point of choosing the next primitive; the next primitive is determinable by a representation of the content in the current position in the sequence.

Among many other sequence structures, *context‐free grammars* can generate center‐embedded sequences in which ordered pairs are embedded within ordered pairs of the same kind. If unbounded (i.e., extendable to arbitrarily many levels of embedding), context‐free grammars require more memory capacity than regular grammars. They require a push‐down stack or computationally equivalent memory architecture (see Fig. 1a). A push‐down stack is a data structure that stores an ordered sequence, A B C …, with a computational architecture that can access that sequence from its *end* only. If you think of this structure as vertically organized, the items are stacked or “pushed” one on top of another, and then can be accessed and removed or “popped” only from the top in a last‐in first‐out order. Items can only be accessed and read after being popped off the stack. Clearly, such a data structure/procedure can easily underlie the production of sequences with center‐embedded structures (e.g., hold ABC in working memory stack, pop off from the top, and immediately read each item popped to create ABC|CBA; see Fig. 1a). Simple center‐embedded sequences of any length could be generated via a push‐down stack, so long as the capacity of the stack is unbounded (e.g., A_1_A_2_B_2_B_1_, A_1_A_2_A_3_B_3_B_2_B_1_, A_1_A_2_A_3_A_4_B_4_B_3_B_2_B_1_ …).

**Fig. 1:**
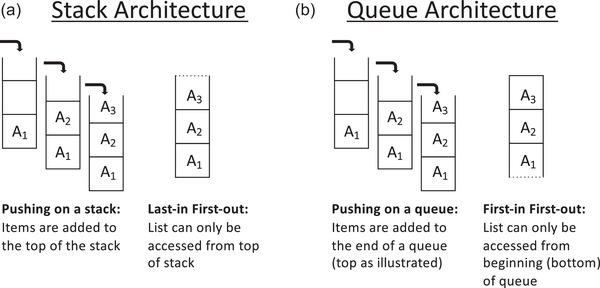
Push‐down stack and queue memory architectures. For both architectures, items are initially stored in the same way (pushed onto a stack or queue). (a) In the stack architecture, the stored list can only be accessed (popped) from the top, creating a last‐in first‐out memory architecture. (b) In the queue architecture, the stored list can only be accessed from the end (shown here at the bottom), creating a first‐in first‐out memory architecture.

Chomsky−Schützenberger contrasted context‐free grammars with *context‐sensitive grammars*, which can generate cross‐serial sequences (e.g., A_1_A_2_A_3_B_1_B_2_B_3_), among other structures. In the Chomsky−Schützenberger analyses, context‐sensitive grammars are more complex than context‐free grammars, requiring memory equivalent to two stacks (see Fig. 2a). An additional stack would be needed to generate these cross‐serial sequences because at the midpoint of the sequence (e.g., A_1_A_2_A_3_ ‐> ?), the matched item needed is subunit 1, but the only accessible item in the stack would be the most recently added, here subunit 3. An additional stack would be needed to reorder the A items so that the item at the bottom of the first stack (A_1_) could be retrieved first.

**Fig. 2:**
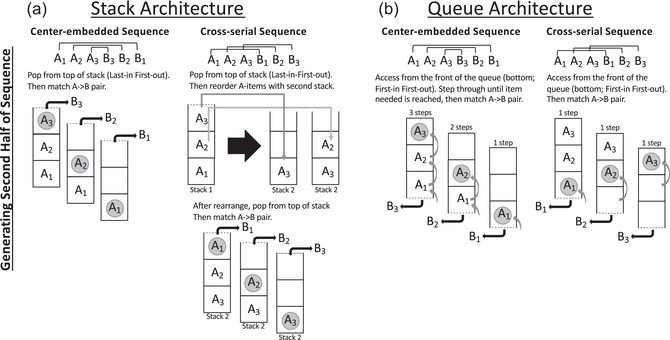
(a) In the stack model, the second half of center‐embedded sequences can just be generated by popping the last item off the stack and matching the A‐>B pairs. Cross‐serial sequences require more processing (and an additional stack) to first rearrange the stack to produce the correct cross‐serial sequence. (b) In the queue model, the second half of center‐embedded sequences requires more processing (iteratively searching through the queue). Cross‐serial sequences can be generated by accessing the first item in the queue and matching the A‐>B pairs.

The Chomsky−Schützenberger work inspired further research that continues to this day. The original work concerned *weak generative capacity* alone—the capacity to generate all and only the allowable sequences in a grammar. Subsequent work concerned *strong generative capacity* as well—the capacity to generate a structural description of the allowable strings (e.g., tree structures; Chomsky, 1965, 1). All work on syntax in formal linguistics seeks strong generative capacity. In the context of weak generativity, the initial Chomsky−Schützenberger distinctions between regular grammars, context‐free grammars, and context‐sensitive grammars have been extended to many dozens of principled distinctions among grammars (among many examples, see, Jäger & Rogers, 2012 for artificial and natural language grammars, Immerman, 2025 for mathematical languages; Icard, 2020 for changes to the Chomsky−Schützenberger hierarchy if grammars are probabilistic rather than deterministic, and Yang & Piantadosi, 2022 for a computational model that learns 82 different grammars, including fragments of natural language grammars, from little data). Although Chomsky explored what grammars underlie the structure of natural languages (e.g., *Syntactic Structures*, 1957), the work on the Chomsky−Schützenberger hierarchy that continues to today concerns not only natural language grammars, but grammars that generate any set of sequences that share an abstract structure. The current work, like most empirical work on artificial grammar learning in psychology (including comparative work), abstracts away from whether the generative mechanisms are implicitly or explicitly represented and concerns weak generativity alone.

Psychological work using artificial grammar paradigms has asked two questions: (1) Are context‐free grammars easier to learn (and center‐embedded sequences easier to produce) than are context‐sensitive grammars (and the cross‐serial sequences they produce)? The issue here is the psychological reality of the complexity hierarchy. This work indirectly bears on the psychological reality of stacks as a psychologically real memory architecture for specific lists. (2) Can nonlinguistic creatures learn grammars that generate all and only center‐embedded or cross‐serial sequences?^2^ Although this second question has led to a rich line of research (Fitch & Hauser, 2004; see Ferrigno, 2022 and Petkov & Ten Cate, 2020 for reviews), including findings used by Chomsky himself to make claims of human uniqueness (Hauser, Chomsky, & Fitch, 2002), we focus here on the first question.

Chomsky did not intend the generative mechanisms articulated in terms of stacks to be psychologically real at an algorithmic level (1957). There are many algorithms that can generate all and only center‐embedded sequences or all and only cross‐serial sequences. The stack architecture abstractly characterizes principled differences in the memory demands involved in generating sequences that satisfy these grammars. Any generative mechanism that generates center‐embedded sequences requires a memory structure *equivalent to* a stack.

Psychological research has compared adults’ ability to learn context‐free grammars that generate center‐embedded sequences and context‐sensitive grammars that generate cross‐serial sequences. This work has found that there is either no difference in the difficulty of learning the two grammars or, more frequently, that context‐free grammars are harder to learn and execute than context‐sensitive grammars (e.g., De Vries, Petersson, Geukes, Zwitserlood, & Christiansen, 2012; Öttl, Jäger, & Kaup, 2015). This has been observed even for natural language grammars. Bach, Brown, and Marslen‐Wilson (1986) made use of the fact that the Dutch and German differ in the structure of clause‐final verb clusters, despite being extremely similar in overall syntactic structure. In Dutch, these sentences have cross‐serial structure, whereas in German and most other natural languages, they have center‐embedded structure.^3^ Bach et al. (1986) found that it was harder for German speakers to compute the correct meaning of center‐embedded sentences than for Dutch speakers to compute the meaning from the semantically identical cross‐serial sentences. This finding is counter to the predictions of the Chomsky−Schützenberger hierarchy and was surprising to many, given that almost all natural languages deploy center‐embedded structures rather than cross‐serial ones in these constructions.

As Janet Fodor is said to have once quipped, “If humans easily deploy stacks, center‐embedded sentences should not be so hard to comprehend.” Indeed, that grammars that generate center‐embedded sequences are, if anything, *harder* to learn and execute than are grammars that generate cross‐serial sequences provides evidence against the psychological reality of stack‐like memory architecture. However, this evidence is not conclusive concerning whether stacks are deployed in the learning and execution of grammars that generate center‐embedded sequences. Stacks may be used, but they may be more difficult to set up and execute than other memory architectures for specific lists. Assessing the psychological reality of stacks requires finding signatures that distinguish stacks from other memory architectures that implement specific lists, such as queues that can be accessed only from the beginning, or whole list representations that can be accessed all at once. We now turn to characterizing the processing signatures of each of these memory architectures.

A queue is a memory architecture that stores a sequence in a *first‐in*, *first‐out manner* (as opposed to a stack's *last‐in*, *first‐out manner*; Fig. 1b). A queue memory architecture can easily underlie the production of cross‐serial sequences (e.g., hold ABC in working memory queue, start from the beginning of list [first in], and immediately read each item from the beginning to create ABC|ABC; see Fig. 2b). However, producing a *center‐embedded sequence* with this type of memory architecture would require additional processing. The stored list, only accessible from the beginning, would be in the reverse order needed—the last item in the queue would need to be accessed first. Thus, the obtained result that cross‐serial sequences are easier to learn and produce than center‐embedded ones is consistent with the hypothesis that queues are being deployed rather than stacks.

The literature on working memory establishes the viability of queues for representing specific ordered lists and provides a further signature of reliance on this memory architecture in online processing. In studies of working memory, it is well‐known that forward recall spans are much longer than backward recall spans (Anders & Lillyquist, 1971; Bireta et al., 2010; Haberlandt, Lawrence, Krohn, Bower, & Thomas, 2005; Hurlstone, Hitch, & Baddeley, 2014). That is, repeating a list in the same order that it was presented (hear “3 8 6 2,” produce “3 8 6 2”) is much easier than repeating it in the backward order “2 6 8 3.” This fact can be understood in terms of the working memory architecture deployed both forward span and backward span tasks being a queue. A queue represents a specific sequence of items in order, first, second, third…, and can *only* be accessed from the beginning. To generate a backward list, one must step through the queue to find the last item, then begin at the beginning again and step through the queue to find the next to the last item, and so on (see the process in Fig. 2b).^4^ This process explains the longer overall response times and higher error rates in the backward relative to the forward span tasks. It also predicts and explains decreasing item‐to‐item response times between successive items as a function of list position in the backward version of the task. As you iteratively search from the beginning of the list, the number of items to step through *decreases* as you get closer to the last item needed (see Fig. 2b, center‐embedded string). This item‐to‐item response time signature has been found in backward‐span memory tasks (see Fig. 3). In contrast, for the forward‐span task, there is no iterative search process; instead, one can just read the items off in the order of the queue, from front to end leading to the observed relatively flat item‐to‐item response times (Fig. 3) Thus, these response time profiles provide signatures that bear on the deployment of stack and queue architectures.

**Fig. 3:**
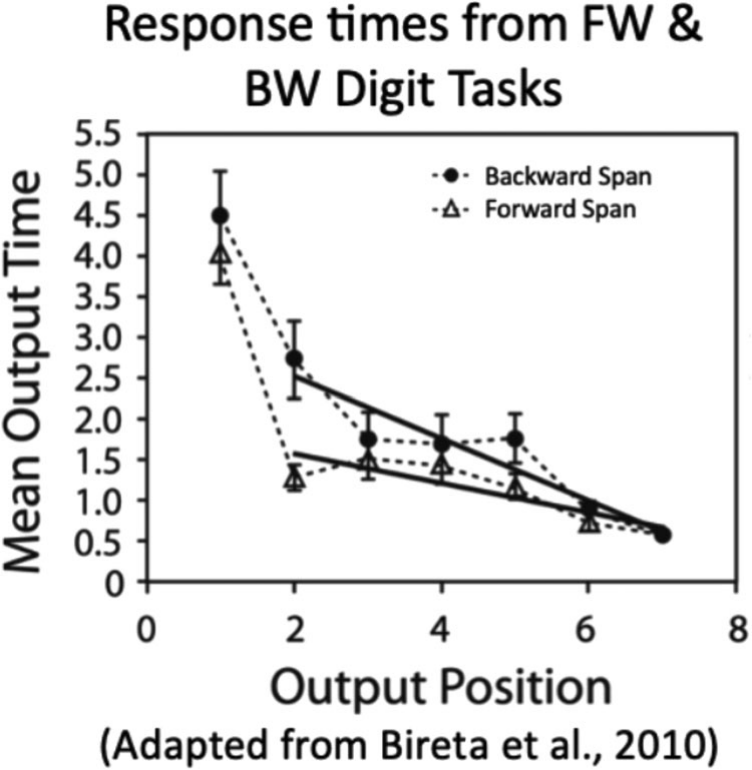
Response times showing the increased processing and a negative slope when iteratively searching through a queue to produce backward orders in the second half of a backward span task (similar to the second half of a center‐embedded sequence). Adapted from Bireta et al. (2010).

Another memory architecture for lists, distinct from both stacks and queues, is a whole list representation in which all items can be accessed at once. A spatialized whole list representation of the sequence of elements A B C D D C B A can be represented in visual working memory. The symmetry in this sequence is perceptually available. Representations of symmetry and other summary statistics of the whole list are signatures of this type of memory architecture, as is content addressability (i.e., being able to judge that D is on the list without stepping through a queue or popping off a stack searching for an item on the list). Working memory for specific sequences, including in natural language processing, is often content addressable, suggesting some type of whole list representation (McElree & Dosher, 1989). Terrace, Son, and Brannon (2003) showed that the overlearned sequences in his sequence productions tasks could be represented in this way; having learned to touch in the order A B C D E F G and presented with B F, monkeys knew to touch B before F, and their response times were shorter than when presented B C. They were not deploying a queue or a stack to plan the sequence of their touches.

Our goal in this study is to establish whether stacks are drawn upon in one of the simplest grammars that generate center‐embedded sequences: an indexed A^n^B^n^ grammar. An indexed A^n^B^n^ grammar not only has dependence between the number of As and Bs as in a standard A^n^B^n^ grammar, but it also contains specific pairs of A‐B item pairs. The positions of the second element of each pair within a sequence are determined by the grammar (e.g., center‐embedded or cross‐serial). That is, the indexes (subscripts) in A_1_A_2_A_3_B_3_B_2_B_1_ specify distinct pairs, and the grammar that generates allowable sequences in such a grammar, such as “1 2 3 3 2 1,” “a b c c b a,” and “( { [ ] } ),” is center‐embedded. A comparable cross‐serial indexed grammar, A_1_A_2_A_3_B_1_B_2_B_3_ with the same base pairs, would generate: A_1_A_2_A_3_B_1_B_2_B_3_, “1 2 3 1 2 3,” “a b c a b c,” and “( {  [ ) } ]” ).

Our target here is ***not*** center‐embedded or cross‐serial structures in natural language. The sequences in our simple indexed grammars do not involve the dependencies that characterize natural language. For one of many examples, in natural language, there is a main clause that not only contains the other clause, but is modified by it—in the sentence “The cat the dog chased ran,” the phrase “the dog chased” is describing which cat ran. In contrast, the sequences tested here and in the previous artificial grammar studies reviewed here do not have this aspect of the hierarchical structure found in center‐embedded natural language sentences. Additionally, our studies, like most in the artificial language literature, concern weak generativity alone. See the General Discussion for many other differences between these artificial grammars and natural language grammars. Furthermore, because they study weak generativity alone, these artificial grammar studies do not address what, if any, hierarchical structure participants have assigned to the sequences that are consistent with the grammar they have induced.

Our work was inspired by the only two artificial grammar studies that have experimentally addressed whether humans use a stack‐like memory architecture to represent center‐embedded sequences (Silva et al., 2022; Uddén, Ingvar, Hagoort, & Petersson, 2012). Both studies tested whether subjects could classify center‐embedded or cross‐serial strings as fitting a given grammar or not. Unlike tasks testing the difference between center‐embedded and cross‐serial sequences, which unfold over time, these tasks presented subjects with the entire strings via a visual presentation (e.g., cross‐serial: “FFDLLP”; center‐embedded: “FFDPLL”) and subjects had to say if each string was grammatical or not. The main prediction from these tasks is that if participants use a stack‐like memory architecture, then center‐embedded strings should be easier to represent (and the ungrammatical strings should be more noticeable) than cross‐serial strings due to the increased processing demands for generating cross‐serial sequences with a stack (see Fig. 2a). However, Uddén et al. found the opposite effect than expected. Participants were more likely to accept ungrammatical strings in the center‐embedded condition compared to the cross‐serial condition (2012).^5^

Following up on this work, Silva et al. (2022) ran a similar task and controlled for repetitions and symmetry. They found that participants in the center‐embedded condition relied on these symmetry cues when they were available. When these symmetry cues were removed, they were unable to reject ungrammatical strings in both conditions (center‐embedded vs. cross‐serial). As mentioned above, sensitivity to symmetry is a signature of memory architectures in which a representation of the whole list is accessible. This suggests that participants represented the entire list using neither a stack nor a queue. Additionally, participants were unable to generate exemplar sequences themselves. Silva et al. did find that participants made a larger number of fixations on nongrammatical strings in the center‐embedded condition compared to the cross‐serial condition. However, given the lack of evidence that participants had learned the grammar, it is unclear how participants represented these sequences. Further research, including evidence that participants have fully learned the grammar, should follow up on the suggestions that stacks are deployed under the presentation conditions of these studies.

Most previous artificial grammar induction tasks use the capacity to distinguish novel grammatical sequences from novel ungrammatical sequences as the measure of having learned the grammar (e.g., Fitch & Hauser, 2004; Gentner, Fenn, Margoliash, & Nusbaum, 2006; Martins, Martins, & Fitch, 2016; Silva et al., 2022; Uddén et al 2012). One problem with this is that above‐chance performance can be achieved with partial knowledge of the grammar. Here, we adapt the Terrace (2005) sequence generation task, which requires subjects to generate complete grammatical sequences. We follow previous work on human and nonhuman grammar induction that has adopted this method (Dedhe, Piantadosi, & Cantlon, 2023; Ferrigno, Cheyette, Piantadosi, & Cantlon, 2020; Liao, Brecht, Johnston, & Nieder, 2022). In this study, the indexed pairs are two identical shapes, with the blue shape preceding the red shape, for example, 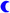 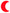 and 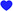 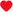. We can thus study when in training participants have learned to produce whole, correctly ordered, novel sequences. Because this task requires sequence production rather than allowable string recognition, we can measure online item‐to‐item production times. This in turn allows us to explore the computational procedures underlying the online production of sequences that satisfy indexed, center‐embedded and cross‐serial A^n^B^n^ grammars.

While one could induce an unbounded indexed A^n^B^n^ grammar from training on such sequences, one might also induce an abstract length‐specific regular grammar, realizable with a finite state automaton. That is, in our study, by the end of training on 4 item arrays, one may have learned (Blue_Shape1_ Blue_Shape2_ Red_Shape2_ Red_shape1_ where shape 1 and 2 are arbitrarily decided, but remain the same across the sequence). This is an abstract grammar to the degree that there is no restriction on what shapes can constitute each pair, but it could be represented with a finite state automaton and would be classified as a regular grammar.

Studies in adult humans have tested whether participants could extrapolate learned grammars to greater depths of embedding (McCoy, Culbertson, Smolensky, & Legendre, 2021; Shin & Eberhard, 2015). After training on sequences with one or two embeddings, A_1_A_2_B_2_B_1_ & A_1_A_2_A_3_B_3_B_2_B_1_, participants were then tested on their ability to evaluate strings with three embeddings: A_1_A_2_A_3_A_4_B_4_B_3_B_2_B_1_. These studies have found that not all participants extrapolated the trained center‐embedded grammar to novel lengths. That some participants could successfully discriminate sequences of novel lengths rules at the same embedding level as training shows all adult participants were *at least* representing the trained sequences with a fixed‐length 4‐item or 6‐item regular grammar. That some participants failed to generalize to new embedding levels suggests that they may have *only* induced length‐specific regular grammars, for these would not generalize to novel sequence lengths. These results show that inducing a simple grammar, either explicitly or implicitly, that expresses the rule with content equivalent to “*put the Bs in reverse order of the As”* is not a trivial matter for participants.

The present experiment, preregistered at https://osf.io/6pe9h/?view_only=0bfcb25ff8a54873836e47353ca10aee, unfolds in several phases. Each phase provides evidence to the participants about the grammar (cross‐serial or center‐embedded). In Phase 1, the participant is trained to correctly sequence two specific 4‐item arrays, which may be learned associatively. In Phase 2, the participant receives a first test on a novel sequence that combines items from the first two lists. This assesses whether associative representations of the two specific training lists are *all* that have been learned, or what grammar, if any, they have abstracted. Phase 3 then introduces entirely novel 4‐item arrays that provide a stronger test for what grammar had been learned. Phase 3 then continues with completely novel arrays of lengths never seen before (i.e., a 6‐item array and then an 8‐item array). This assesses whether participants have learned only a length‐specific regular grammar in Phases 1 and 2. The novel test arrays are not error‐corrected; any sequence order is accepted, so it is possible to infer what grammar (at the level of weak generativity) the participant has abstracted by that point in the experiment. Finally, we analyze the item‐to‐item response times between successive touches in the second half of correctly sequenced novel arrays to assess whether participants are deploying stacks in the generation of center‐embedded and cross‐serial sequences.

## 1 Methods

### 1.1 Participants

Participants were 100 adults (*mean age: 31*, *SD: 8.4*, *68 male)*. Half were randomly assigned to the center‐embedded condition and half to the cross‐serial condition. All participants were recruited from Amazon Mechanical Turk and paid $3 for participation. Procedures for recruitment and the experiment itself were approved by the Harvard University Research Subjects Review Board.

### 1.2 Procedure

**Fig. 4:**
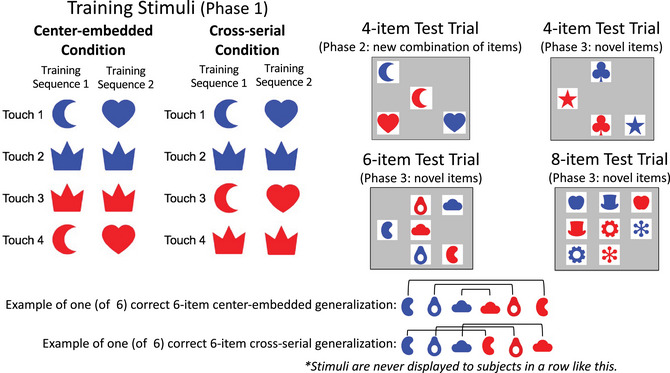
Examples of each trial type. The training stimuli (Phase 1) consisted of two center‐embedded or cross‐serial lists, depending on condition. After training, participants received the “4‐item Test Trials” (Phase 2), which consisted of a novel combination of items from the training list that were presented without feedback. After Phase 2, participants were given five trials of the 4‐item test trials without reinforcement. They were then given additional training trials on the novel array (with feedback), before moving to the next array size. For each of the arrays, there are multiple correct answers. One correct sequence of touches for each condition is illustrated for the 6‐item array length. Participants never saw arrays with the correct order arrayed in space.

Participants completed the entire task on their home computers while in full‐screen mode. The experiment began with 4‐item arrays. Participants were instructed that the goal of the task was to click on images in the correct order and that they must learn this order through trial and error. Throughout the whole experiment, all training trials began by clicking the start stimulus, a small robot. Then, four pictures were displayed randomly distributed within a box outlined on their monitor (see Fig. 4 for examples of arrays). When a picture was clicked, it gave auditory feedback (a ding) and visual feedback (briefly fading for .2 s) to cue that the click was registered. Throughout a trial, all items remained on the screen after being clicked, thus remaining selectable. Participants then moved onto the next choice until all items had been selected in the correct order, or until an error had been made. If an error was made on a feedback trial, participants received negative auditory feedback (a buzz), a blank face image, and a 2‐s time out screen immediately after the incorrect touch, before a new trial was initiated. A new array was then presented (with different spatial arrangements of the images). If a participant completed the trial correctly (i.e., clicked on all items in the correct order), they heard positive auditory feedback (chimes) and saw a happy face to indicate that the trial was correct. After a correct trial, participants immediately moved onto the next trial.

The training stimuli (Phase 1) consisted of two center‐embedded or cross‐serial sequences, depending upon condition. After training, participants received the “4‐item Test Trials” (Phase 2), which consisted of a novel combination of items from the training list that were presented without feedback. After Phase 2, participants were given the five novel 4‐item test trials without reinforcement. They were then given additional training trials on the same novel array (with feedback), before moving to the next array size. The testing of generalization to new novel arrays with no feedback, and then further training on these novel arrays with feedback, was then repeated at new sequence lengths (6 and 8). For each of the novel arrays, there are multiple correct answers, depending upon how the participant chose to order the first half of the sequence (the blue shapes). Most participants (in both conditions) produced more than one correct sequence for at least one array size. One (of six) correct sequence of touches for each condition is illustrated for the 6‐item array length in Fig. 4.

### 1.3 Stimuli

The stimuli consisted of paired blue and red simple shapes (see Fig. 4). The color of the shapes determined the class within the indexed A^n^B^n^ grammar (blue = A items, red = B items). The shape of the image determined the base pair unit (expressed by subscript values) within the indexed A^n^ B^n^ grammar (e.g., in the first training sequence, A_1_ & B_1_ were both moons, one blue and one red). These two dimensions (color & shape) allowed us to create the indexed center‐embedded (and cross‐serial) sequences, such as the center‐embedded sequence: A_1 [Blue Moon]_, A_2 [Blue Crown],_ B_2 [Red Crown],_ B_1 [Red Moon]_. Here, the crown unit (A_2_ B_2_) is embedded within the moon unit. In the cross‐serial condition, the A_2_ B_2_ shapes were also crowns in both training sequences, and the A_1_ B_1_ shapes were also moons in the first sequence and hearts in the second sequence.

### 1.4 Conditions

Participants were randomly assigned to one of two conditions (center‐embedded or cross‐serial). In the center‐embedded condition, participants were trained to click the images in a center‐embedded sequence (e.g., A_1_A_2_B_2_B_1_, where the pair of shape 2 was embedded within the pair of shape 1; see Fig. 4, Training Stimuli). In the cross‐serial condition, participants were trained to click the images in a cross‐serial sequence (e.g., A_1_A_2_B_1_B_2_, where the pairs are interleaved; the correct order of the B items was the same as the A items; see Fig. 4, Training Stimuli).

### 1.5 Phase 1: Initial training trials

Participants were trained on the correct sequence of touches for the first training array, followed by learning the sequence of touches on the second training array. There was one correct sequence of touches for each array. The order of touches of the blue items was arbitrarily decided and had to be discovered by trial and error, but the order of touches of the red items was computable from the grammar of each condition. Participants were required to correctly sequence the first array in four out of five trials in a row before they moved onto the second initial training array. As can be seen in Fig. 2, the second array included one base pair that was the same as in the first array (the crowns). The blue item from this base‐pair was the second item touched in both arrays, that is, in both sequences, the second item touched was the blue crown. The purpose of having two arrays was to introduce participants to the idea that there was a common grammar that applied to the correct sequencing of touches on different arrays.

### 1.6 Phase 2: 4‐item test trials (novel combination of trained items, no feedback) combined with further training trials from Phase 1

After completing both training arrays, participants moved to test trials with a novel array consisting of four items that had not appeared together in a single array before (the hearts and moons of Array 1 and Array 2, respectively; see Fig. 4, 4‐item Test). There were two correct sequences of touches on the novel test array (e.g., in the center‐embedded condition: “Blue Heart ‐> Blue Moon ‐>Red Moon ‐>Red Heart” OR “Blue Moon ‐> Blue Heart ‐> Red Heart ‐> Red Moon”), and there were similarly two correct sequences of touches in the cross‐serial condition.

One goal of these test trials was to rule out that participants had learned each of the 4‐item arrays as two independent, arbitrary sequences, not noticing the regularities due to color order or repetition orders of shapes. Research on list learning with monkeys shows that if taught two independent lists (A B C D) and (E B C F) and then tested on arrays that include A D E and F, monkeys produce the items that had come first in each of the previous lists first and those that had come last in each of the previous lists last. Having just learned this item‐specific information would yield correct orders on the novel combination test arrays half of the time in each of the conditions (Ferrigno et al., 2020). And having only learned the partial grammar (*touch blue before red)* would also predict half‐correct orders on the novel combination test arrays. Thus, comparing the proportion of correct sequences on the novel combination test arrays to 50% will assess whether participants memorized only the specific sequences of shapes for the two training arrays, as well as whether they had abstracted a more complete grammar than one that merely generates sequences where both blue shapes precede both red shapes.

Participants received positive feedback regardless of their accuracy on these test trials; that is, they were not corrected if they failed to produce a sequence that satisfied the center‐embedded or cross‐serial grammar they had been trained on. Thus, these test trials did not provide any new evidence to the participants about the correct grammar, as the novel combination arrays were never differentially reinforced. The test trials were intermixed with further reinforced training trials from the original training arrays (Fig. 4, Training Stimuli). These additional training trials were used to provide further training on the original arrays, to ensure that the original training was maintained, and to help participants remember the order within each of the trained arrays. Again, these trials provided no new information about the correct grammar. Participants received 20 total trials (10 4‐item novel combination test trials [no feedback] and 10 additional training trials, 5 using each training array).

### 1.7 Phase 3: 4‐, 6‐, and 8‐item test trials (test arrays with completely novel stimuli)

Our experiment was designed to explore the difference in difficulty of inducing center‐embedded and cross‐serial grammars from little information (see Fig. 2). Over Phases 1 and 2, participants had received error feedback on only *two* sequences that satisfied the grammar in their condition grammar—the training sequences shown in Fig. 4. In Phase 3, participants were tested with arrays constructed from stimuli they had never seen before. The images had the same color structure (blue and red), with color determining the ordering within a unit and a unit consisting of two identical‐shaped pairs as before (e.g., A_1_ B_1_; see Fig. 4). The shapes in the novel test trials were new. The novel 4‐item array test trials assessed whether participants had induced a grammar that abstracted beyond the two training arrays. They tested for, at least, a fixed‐length regular grammar with abstract variables (i.e., A_1_A_2_B_2_B_1_ for center‐embedded 4‐item sequences and A_1_A_2_B_1_B_2_ for cross‐serial 4‐item sequences), or, equivalently, a grammar like *first all the blues*, *then all the reds; make the first and last item the same shape*. A grammar with that content would generate grammatical 4‐item sequences but would not generalize accurate sequencing to the 6‐ or 8‐item array.

The 6‐ and 8‐item arrays were designed to test whether participants had learned abstract A^n^B^n^ center‐embedded and cross‐serial grammars that generalized to additional levels of embeddings. There were many different correct sequences for these 6‐ and 8‐item sequence trials. Participants had to first decide on an order to touch the blue stimuli (A items) and then had to implement a procedure that produced either the reverse of that order (center‐embedded condition) or a repeat of that order (cross‐serial condition). Any sequence that was correctly center‐embedded or cross‐serial (depending on the condition) was treated as correct, regardless of how participants ordered the blue items.

For each array size in Phase 3, participants first received five nondifferentially reinforced test trials with never‐before‐seen shapes. These trials provided evidence about what grammar had been induced up to that point. Then, starting on trial 6 for each novel array, participants received additional training trials (with feedback) on the *same* novel array. These feedback trials were used to test if participants could learn the correct sequences licensed by the given grammar for that particular array size, even if they had not done so already. This also allowed us to measure the difficulty of implementing each type of sequence. These trials thus provided the first new evidence beyond that received on the training arrays as to what the target grammars were. Participants received a total of 25 4‐item trials (5 test trials with no feedback and then 20 training trials with error correction) and 45 6‐ and 8‐item trials (5 test trials with no feedback and then 40 training trials with error correction for each array size). The error correction during training trials was the same as in the initial training, except that there was no constraint on the order of the initial blue items. That is, if participants did not touch 4 unique blue items first (i.e., if they included a red item in the first 4 touched, or touched a particular blue item twice), or if they touched a single red item out of order according to their condition, they heard the error signal and had a 2‐s time out before the next trial began.

### 1.8 Exclusion criteria

As specified in the preregistration, we excluded outliers for each analysis (individuated by condition, unreinforced generalization test trial, reinforced training trial, and array size). No participants were excluded from the entire study. Instead, participants’ responses were excluded from specific analyses based on preset criteria. See Supplementary Materials for the preset exclusion criteria, as well as the number of participants whose data were excluded from each analysis. This allowed us to include as many participants as possible while removing any outlying data points that would drastically increase variability and noise in the data. As specified in the preregistration, we also excluded some specific trials if there were indications of inattention within that trial (trials where the same item was pressed >2 times). As expected, the exclusion of data as described in the Supplementary Materials decreased noise. The Supplementary Material also reports qualitatively identical results, with respect to the effects interpreted here, when no outliers were removed.

## 2 Results

### 2.1 Overview of analytic approach

All data processing and analyses were conducted using statistical packages in R. The main preregistered analyses reported the use of linear mixed‐effects models fit using the lme4 package. We initially included the maximal random effects structure for each model (Barr, Levy, Scheepers, & Tily, 2013). If the data could not support the model, we removed random effects terms until the models converged. We analyzed both error data and Response Time data with these models. To compare between condition and lengths, we computed post hoc Tukey‐adjusted pairwise comparisons using the emmeans package (Lenth, Singmann, Love, Buerkner, & Herve, 2019). Although the main analyses use linear mixed‐effects models, as specified in the preregistration, we also used one‐sample *t*‐tests to compare participants’ performance to chance, and to what would be achieved by associative learning or the partial grammar “touch blue before reds,” which change based on the length of the sequences. These *t*‐tests provide an initial rough estimate of the success of participants in each condition and the list length at which they have learned some grammar that generates the correct sequences. Lastly, we use a Bayesian mixture model to test two possible memory architectures that might underlie these grammars, stacks, and queues, described in Section 4, Computational Models.

### 2.2 Phase 1: Training results

It was equally easy for participants in each condition (center‐embedded, cross‐serial) to learn to touch in sequence the individual items of two different 4‐item training arrays. On average, participants took 14.1 (*SD* = 9.2) trials to reach the criterion on the first array, and 6.1 trials (*SD* = 2.4) to criterion on the second array. A series of linear mixed model examined the effects of array presentation order (first, second), condition (center‐embedded as the base condition), their interaction, and a by‐subject random intercept (the maximal structure that was not singular) on the DV trials to criterion. We found that the best fitting model included array presentation order (*χ* ^2^(1) = 55.03, *p* < .001; compared to the null model). Including condition in addition to array presentation order did not improve the fit (*χ* ^2^(1) = 1.41, *p* = .23). Participants in each condition learned the first two training sequences equally easily. There was a main effect of array presentation order, which reflected faster mastery of the correct sequence of touches for the second array (β_ArrayOrder_ = −8.06, Wald 95% CI = [−10.01, −6.10], *t*(87)= −8.06, *p* < .01; see Supplementary Materials for random effects and full model results).

The improvement from the first to the second array could be due to having induced at least a partial grammar from just one encounter with a specific sequence. Alternatively, the 4‐item sequences in both conditions might have been learned as arbitrary sequences (A B C D) and (E B C F), where B and C were blue and red crowns, without extracting the underlying grammar. Associative models of arbitrary sequence learning (e.g., Terrace, 2005) could accommodate the effect of array presentation order, since two specific items were identical, and in the same sequencing position, across the two training arrays. The novel combination test trials adjudicate between these two possibilities.

### 2.3 Phase 2: 4‐item test trials, novel combination of base pairs

The percentage of completely correct sequences in the test trials was compared to that expected from random responding (8.3%), and to what would be expected if participants had merely learned the generalization “touch blue, and then touch red” (50%) or were merging average position of the exact stimuli of the two lists (also 50%). To compare performance in each group against the proportion correct, we would expect to see if subjects were deploying associative representations of the specific lists or a partial grammar. We ran one‐sample *t*‐tests comparing subjects’ performance to the predicted accuracy on either of them (50%) for each condition. Participants performed much better than if all they had learned was a Blue then Red grammar or if they were deploying an associative ordinal strategy, each of which would have generated correct sequences at 50% of the time (one‐sample *t*‐tests, two‐tailed: Center‐embedded: 77% correct sequences, *t*(49) = 5.76, *p* < .001; Cross‐serial: 91% correct sequences, *t*(49) = 16.05, *p* < .001, see Fig. 4). They represented the sequences with a grammar that was abstract enough to produce correct sequences from novel arrays that combined items from distinct training arrays. As in Ferrigno et al. (2020), which used brackets as stimuli (e.g., “{[]}”) and included only center‐embedded sequences, participants were able to generalize the two 4‐item sequences to novel combinations of items. Success in the present study shows that adults do not require experience with mathematical or logical formalisms in the form of brackets to easily learn a grammar that generates center‐embedded sequences with one level of embedding.

**Fig. 5:**
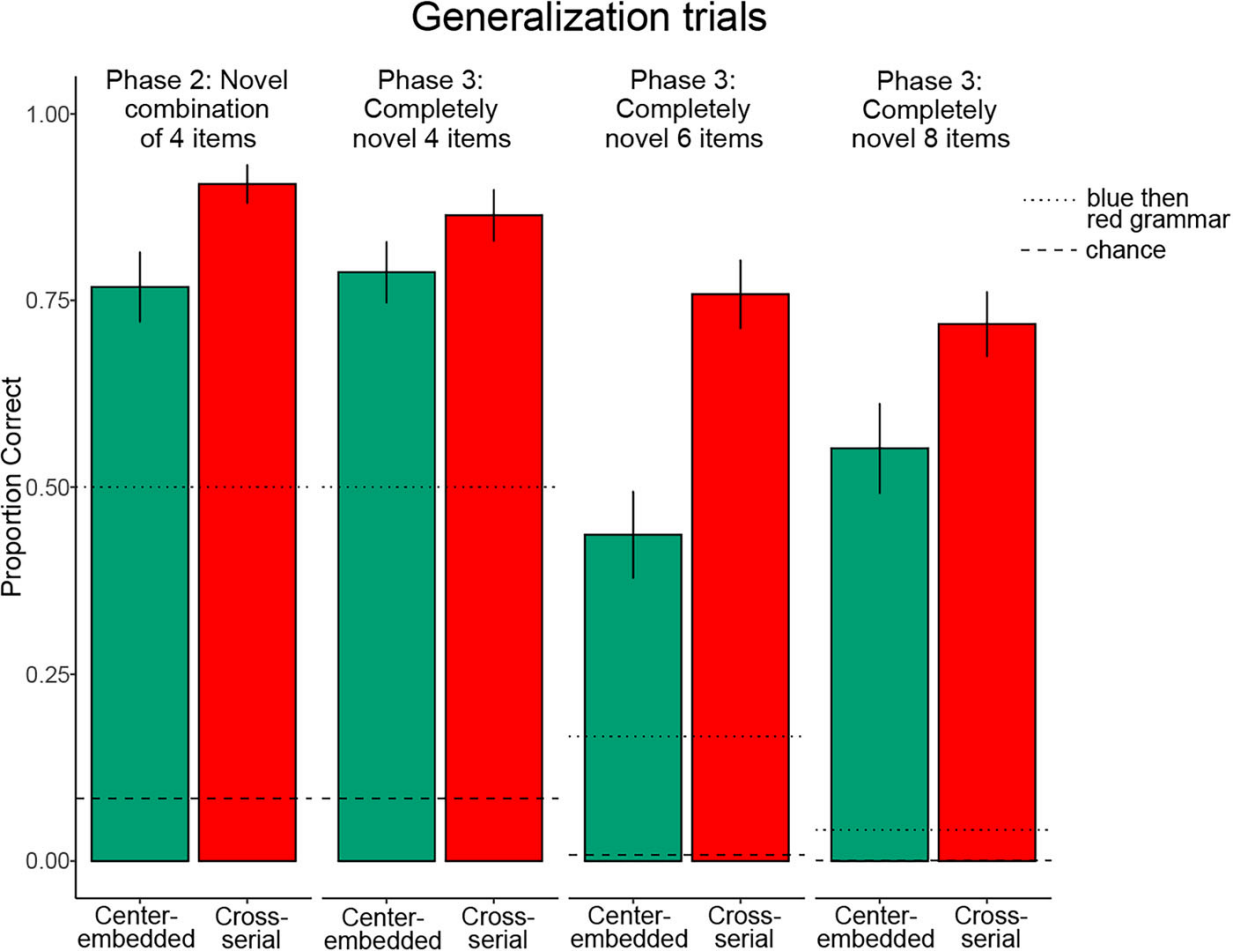
The proportion of fully correct sequences in the 4‐item test trials from Phase 2 (novel combination of base pairs from the training arrays) and Phase 3 (completely novel items) in each condition and array length. Error bars represent the standard error of the mean. The dotted line represents predicted accuracy from a blue then red grammar (i.e., disregarding the base pairs), and the dashed line represents chance (random responding).

While participants in both conditions successfully sequenced the novel test arrays better than 50% of the time, performance was better in the cross‐serial condition than in the center‐embedded condition (see Fig. 5). We used a mixed effects logistic regression to quantify the effects of condition (center‐embedded or cross‐serial) on the number of correct generalizations using condition as a fixed effect and a random intercept of participant. The inclusion of a random slope of condition by participant produced a singular fit and was removed. We found that the best fitting model included condition (*χ* ^2^(1) = 5.33, *p* = .021, compared to the null model). Subjects were more likely to touch the images in the correct order in the cross‐serial condition (β_condition_ = 5.42, Z = 2.32, *p* = .003). The cross‐serial grammar was easier to learn, or the online implementation of the cross‐serial order sequences was easier (and thus led to fewer errors), or *both* learning and online execution of cross‐serial sequences were easier than that of center‐embedded sequences. Previous observations that center‐embedded indexed A^n^B^n^ grammars are as hard or harder to learn and/or to execute than are cross‐serial ones (Bach et al., 1986; De Vries et al., 2012; Öttl et al., 2015) were confirmed here, in this extremely early stage in the process. This pattern is seen after just *two specific* grammatical training sequences, in generalization to a new sequence that partially overlaps with the training ones.

### 2.4 Phase 3: Test trials. Completely novel 4‐, 6‐, and 8‐item arrays

The data on the Phase 2 test trials show that from experience with just two training arrays, arrays that had some common shapes in the same positions were sufficient for adults to abstract a grammar that went beyond *touch blue shapes before red shapes*. Phase 3 test trials tested whether participants could generate correct sequences of touches on arrays that contained red and blue items with shapes not used in training (see Fig. 3: star and club for 4‐item arrays; bean, avocado, cloud for 6‐item arrays; apple, hat, snowflake, gear for 8‐item arrays). No associative process operating over the two memorized lists that were reinforced during training would generalize to complete sequences using novel shapes.

#### 2.4.1 Novel 4‐item array test trials

The novel 4‐item test trials tested whether the very minimal training in Phases 1 and 2 led to grammars that were at least equivalent to a fixed‐length regular grammar stated in abstract variables. If subjects have abstracted a grammar that generates the relevant 4‐item sequences, performance on the 4‐item novel array test trials (involving blue and red stars and clubs) at least should be above 50%, as we found in the 4‐item novel combination test trials of Phase 2. As seen in Fig. 5, which displays the proportion of correct sequences in the five unreinforced (i.e., no error feedback) test trials, this is what was observed. Participants in both conditions produced correct sequences on the novel 4‐item arrays more than would be expected by chance, and more than would be expected if they had only learned that they should touch blue items before red ones, which would generate correct sequences 50% of the time in each condition (blue then red strategy = 50%, Center‐embedded: 79%, *t*(48) = 7.09, *p* < .001; Cross‐serial: 86%, *t*(45) = 10.70, *p* < .001). Additionally, in the later section, we confirm this result in a mixed effects logistic regression predicting accuracy using condition and array size as predictors.

As can be seen from Fig. 5, performance on the novel 4‐item sequences in the Phase 3 test trials was essentially identical to that on the novel combination lists of Phase 2. This result is important, for it shows that feedback on just two training trials was sufficient for participants to induce an abstract grammar with at least the expressive power of a length‐specific regular grammar (i.e., equivalent to center‐embedded: A_1_A_2_B_2_B_1_, cross‐serial A_1_A_2_B_1_B_2_).

#### 2.4.2 Novel 6‐item array test trials

Performance on the five novel 6‐item arrays with no error correction assessed whether the grammar participants in each condition had abstracted by the end of training trials of the new 4‐item list from Phase 3 would generalize to an additional level of embedding. The novel 6‐item array was the first array of this length that the subjects had encountered in the experiment.

As seen in Fig. 5, performance on the 6‐item test trials in both conditions was better than would be expected by chance (0.8% correct) and better than would be expected if participants had learned only that they should touch blue items before red items (17% correct): Center‐embedded: 44%, *t*(48) = 4.69, *p* < .001; Cross‐serial: 76%, *t*(47) = 13.05, *p* < .001. At least some participants, but certainly not all, had learned a grammar from the training on 4‐item arrays that generalized to one further level of embedding. This is a conceptual replication of the findings of Shin and Eberhard (2015) and McCoy et al. (2021).

The great decrease in correct sequencing from the 4‐item novel array test trials to the 6‐item novel array test trials shows that not all participants generalized the grammars acquired during the 4‐item training to new levels of embedding. A large proportion of participants had not induced a grammar equivalent to *touch th*e *red shapes in reverse (center‐embedded sequences)* or *same order from the order of the corresponding blue shapes (cross‐serial sequences)* by the end of the training on the novel 4‐item sequences. If participants had learned this rule and represented it explicitly, they should be at ceiling on this task (list lengths of 3 and 4 blue shapes are well within the forward and backward working memory spans of adults). The observed decline would be expected if some participants had learned only a fixed length abstract grammars from their 4‐item training. As shown in the logistic regression reported below, this was true for the center‐embedded condition but not the cross‐serial condition.

#### 2.4.3 Novel 8‐item array test trials

The novel 8‐item array was the first array of this size that participants had ever encountered. Performance on the five unreinforced test trials assessed whether participants had learned grammars that would generalize to an additional level of embedding, at least after error‐corrected training with both 4‐item (one level of embedding) and 6‐item (two levels of embedding) sequences. As seen in Fig. 4, performance in both conditions was vastly better than would be expected if participants had learned nothing that would generalize to 8 items (chance 0.006%) or only that they should touch blue items before red items (4% correct): 8‐item arrays: Center‐embedded: 55%, *t*(44) = 8.58, *p* < .001; Cross‐serial: 72%, *t*(48) = 15.80, *p* < .001. Furthermore, the drop in performance observed between 4‐ and 6‐item arrays was not seen between 6‐ and 8‐item arrays, in either condition (see regression analysis below). This suggests that the success on 6‐item arrays by the end of error correction training was not due to having learned new fixed length regular grammars but was subserved by an A^n^B^n^ grammar that generalized to a new level of embedding. However, performance was far from at ceiling, and as we next show, performance was significantly better on the cross‐serial sequences than on the center‐embedded ones.

#### 2.4.4 Preregistered logistic regression

A series of logistic regressions examined the effects of condition (center‐embedded vs. cross‐serial), array size (4‐item, 6‐item, 8‐item), the interactions between these variables on the dependent variable, probability of a completely correct sequence. We used random effects terms that included by‐subject random intercepts, and by‐subject random slopes for condition and array size. The best fitting model included fixed effects for condition, array size, and their interaction (*χ* ^2^(2) = 8.045, *p* = .018, compared to a model without the interaction term). Inclusion of a by‐subject random effects term of interaction did not improve the model fit and was not included (*χ* ^2^(11) = 2.055, *p* = .99; see Supplementary Materials for complete model details and random effects). The goal of this analysis was to explore whether cross‐serial grammars were easier to learn than center‐embedded grammars, as would be expected if participants used queues rather than stacks to implement the indexed A^n^B^n^ grammars that were eventually induced.

To assess the difference between conditions, we first ran a pairwise comparison between the center‐embedded and cross‐serial conditions, collapsed across array length. Participants in the cross‐serial condition were more likely to produce correct sequences of touches than were those in the center‐embedded condition (β_condition_ = −1.13, Z = −2.72, *p* = .003).

To assess whether there was a cost of increasing array length, we ran pairwise comparisons between each of the three array sizes. Simultaneous pairwise comparisons using Tukey's HSD test indicated that accuracy was significantly better in the 4‐item arrays than in the 6‐item arrays (Pairwise Comparison: β_4‐item vs. 6‐item_ = 1.95, Z = 5.15, *p* < .001) and on the 4‐item arrays than on the 8‐item arrays (Pairwise Comparison: β_4‐item vs. 8‐item_ = 1.74, Z = 4.57, *p* < .001), and there was no significant difference between the 6‐item arrays and the 8‐item arrays (Pairwise Comparison: β_6‐item vs. 8‐item_ = −.21, Z = −0.209, *p* = .94). This confirms that not all participants spontaneously generalized from 4‐ to 6‐item arrays, and that after training on 6‐item arrays, whatever grammar the participants had abstracted generalized to an additional level of embedding.

Furthermore, we found a significant interaction between condition and array size in the 4‐ versus 6‐item array size comparison (β_condition * 4‐item vs. 6‐item_ = 1.58, Z = 2.07, *p* = .03), but no significant interaction between condition and array size in the 4‐ versus 8‐item array size comparison (β_condition * 4‐item vs. 8‐item_ = .05, Z = 0.07, *p* = .94). That is, when the array size increased from 4‐ to 6‐items, performance decreased more in the center‐embedded condition relative to the cross‐serial condition (see Fig. 4). In the center‐embedded condition, performance fell drastically from the 4‐item array (79% correct) to 6‐item array (44% correct). To test if this difference was significant and compare it to changes in the cross‐serial condition and the 6‐ to 8‐item change, we ran a series of post‐hoc pairwise comparisons using Tukey's HSD test. We found that there was a significant decrease in correct sequences between the 4‐ and 6‐item length in the center‐embedded condition (Pairwise Comparison: β_Center‐embedded: 4‐item vs. 6‐item_ = 2.73, Z = 5.65, *p* < .001). We found no significant change between the 4‐ and 6‐item length in the cross‐serial condition (Pairwise Comparison: β_Cross‐serial 4‐item vs. 6‐item_ = 1.15, Z = 1.97, *p* = .36). That performance fell from the 4‐item novel arrays to 6‐item novel arrays in the center‐embedded condition is consistent with the possibility that some participants in the center‐embedded condition may have initially represented the grammars in the 4‐item arrays in terms of a fixed length regular grammar.

As an exploratory analysis, we analyzed the errors produced during the five test trials with no error feedback in each condition. In the 6‐ and 8‐item center‐embedded trials, we found a large portion of errors where all “A” items are pressed first and “B” items second, and a mismatched pairing of the base items (e.g., A_1_A_2_A_3_B_2_B_1_B_3_). Although these types of errors were found for both grammars, they were much more frequent in the center‐embedded condition (6‐item array: center‐embedded: 38%, cross‐serial: 13%, 8‐item array: center‐embedded: 32%, cross‐serial: 16%). Furthermore, in the center‐embedded condition, there were also many responses that were fully cross‐serial orders (6‐item: 16% crossed and 8‐item: 12% crossed), but the reverse was not seen in the cross‐serial condition (<1% in both 6‐ and 8‐item conditions).

One interpretation of the entire pattern of results is that for *some participants*, especially in the center‐embedded condition, further learning of the grammar underlying the correct sequence of touches beyond training on 4‐item arrays is required to generalize the grammar to 6‐ and 8‐item arrays. Suppose for some participants the abstract grammar learned in the 4‐item training trials in the center‐embedded condition was Blue_Shape1_Blue_Shape2_Red_Shape2_Red_Shape1_, a length‐specific regular grammar representation. This does not generalize to the 6‐ or 8‐item lists. The learner could then analyze this representation to seek hypotheses relevant to the larger arrays. A first obvious hypothesis is that blues proceed red. That hypothesis is reflected in the high level of sequences that satisfy only this generalization in the center‐embedded novel array 6‐ and 8‐item test trials. A second obvious hypothesis is that there is something systematic about the order of the blues that determines the order of the reds, and the cross‐serial systematicity is a more salient hypothesis than the center‐embedded one. This is reflected in the high level of fully cross‐serial sequences among participants on the center‐embedded novel array (6‐ and 8‐item) test trials.

The data presented so far are consistent with the hypothesis that the center‐embedded grammar is more difficult to learn than is the cross‐serial grammar. Being based on generalization error data alone, they are also consistent with the hypothesis that even after an unbounded indexed center‐embedded and cross‐serial grammar is learned, the online process used to generate center‐embedded sequences is harder to execute than for center‐embedded sequences. In Section 3.5, we turn to the response time data to explore the latter hypothesis. In Section 4, we will present a computational model that separates errors due to not having learned the grammar from errors due to difficulty producing the sequences.

We now turn to the question of what computational machinery is used to implement the correct grammar to generate sequences that are consistent with it.

### 2.5 Response time analyses (correct sequences in the further training trials: 6‐ and 8‐item arrays)

The above analyses reveal that participants made more sequencing errors on novel test trials in the center‐embedded grammar than in the cross‐serial grammar. If the above results reflect differences in the difficulty of generating each type of sequence, we should observe overall response time differences that persist once the correct grammars are learned. Such a finding would be consistent with queues being used to produce both sequence types. However, it is also possible that queues are used to produce cross‐serial sequences and stacks are used to produce center‐embedded sequences, and that stacks are harder to use than queues.

We can adjudicate between these two possibilities by comparing item‐by‐item response times between successive touches on the second half of the list (the red items) in the 6‐ and 8‐item sequences. A stack memory architecture would predict flat item‐to‐item response times for the second half of center‐embedded sequences. If stack architecture is being deployed in both conditions, there should be a longer delay for the first item in the second half of the list in the cross‐serial condition. Stack architecture would predict a longer delay for the first item in the second half of cross‐serial sequences than in center‐embedded sequences, because the items in the initial stack would need to be popped off and added to a second stack to reverse the order. For the same reason, their stack architecture would predict overall longer response times for the cross‐serial sequences than center‐embedded ones. In contrast, if queue architecture is being deployed in both conditions, we should see a negative slope for the item‐to‐item response times in the second half of center‐embedded sequences, a relatively flatter slope for cross‐serial sequences, and overall longer response times for center‐embedded sequences (see Fig. 3).

Here, for the first time, we analyze data from the further training trials, the part of the study following the test trials with no error feedback. These further training trials provide error feedback on participants’ sequencing of the novel 4‐, 6‐, and 8‐item arrays. We only included trials where subjects produced a complete correct sequence of touch trials. We excluded the time between the last blue item and the first red item (e.g., the response time between item 3 and item 4 in the 6‐item list) because these response times could reflect the time to start producing the second half of the list (e.g., the time to switch from pushing items onto a stack to popping them, the time to begin searching through a queue, or the time for encoding the red shapes in preparation for executing the grammar). For this reason, we did not analyze the 4‐item novel sequences.

#### 2.5.1 6‐item sequences

**Fig. 6:**
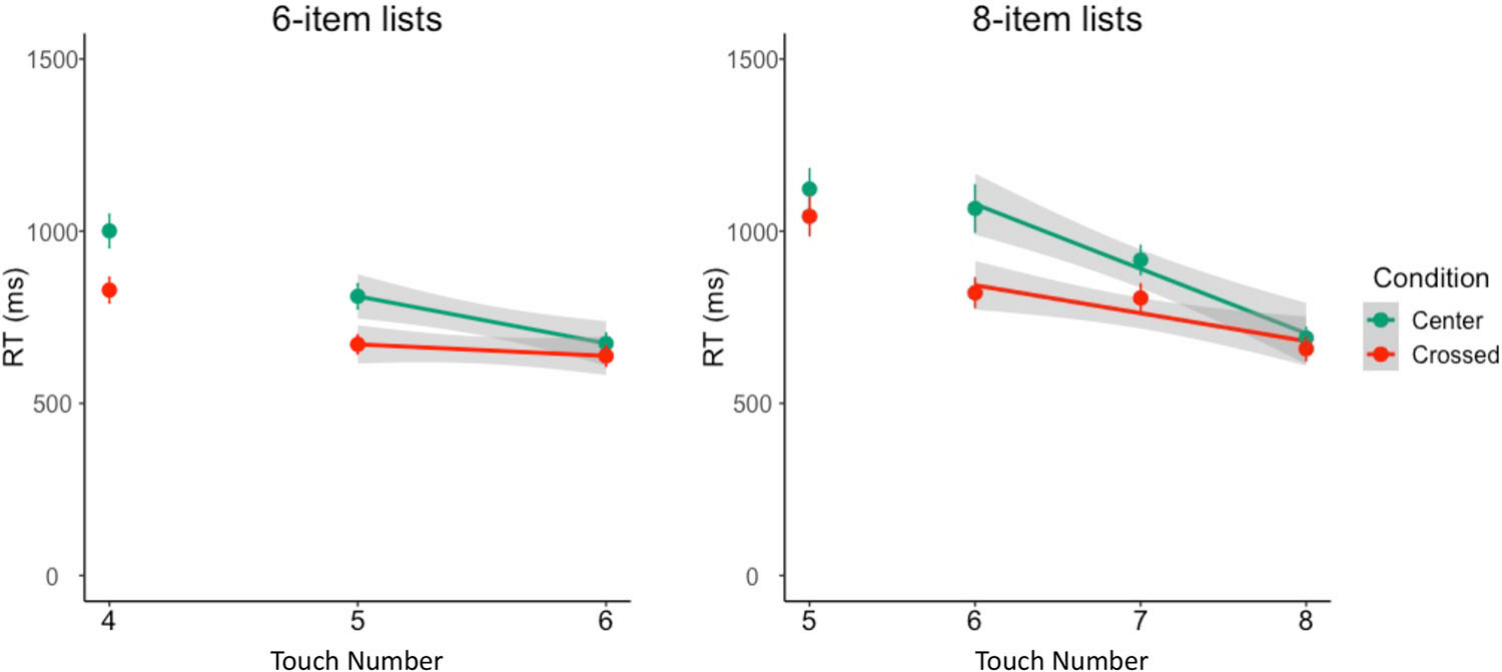
Response times between touches in the second half of the sequences. That is, mean RTs (points) and model estimates (lines) between third touch and fourth, the fourth and fifth, and the fifth and sixth on the 6‐item sequence list, and mean RTs (points) and model estimates (lines) between the fourth and fifth, fifth and sixth, sixth and seventh, and seventh and eighth touches on the 8‐item sequence. Error bars represent the SE of the mean, and the shaded region represents the 95% confidence interval of the model.

We ran a preregistered mixed effects linear regression examining the effects of condition and touch number on *item‐to‐item response time* and *overall (summed) item‐to‐item RTs* for the second half of the list (excluding the first item). We also included the maximal random effects structure when possible. However, the full model, including a by‐subject random interaction term, did not converge, and random effects terms were removed until the model converged (see Supplementary Materials for complete model details). We found that the best fitting model included fixed effects of both main effects and an interaction term (*χ* ^2^(1) = 13.49, *p* < .001). Overall, the second half of the center‐embedded sequences took longer to produce than cross‐serial sequences in the 6‐item arrays (β_condition_ = −640.8, *t*(81.8) = −4.07, *p* < .001; see Supplementary Materials for full model description and results including random effects). We also found a main effect of touch number, such that in the base condition (center‐embedded), item‐to‐item response time decreased for successive touches as each trial progressed (β_touch_ = −136.7, *t*(83.6) = −4.07, *p* < .001). Lastly, we also found a significant interaction between condition and touch number such that the center‐embedded condition had a steeper item‐to‐item response time slope compared to the cross‐serial condition (see Fig. 6; β_condition*touch_ = 100.4, *t*(80.7) = 3.77, *p* < .001). To make sure this effect held when including just the participants who had learned the grammar, we also replicated these results using only participants who produced correct sequences more than 80% of the trials in the 40 error‐corrected test trials and found qualitatively similar results (see Table S14).

#### 2.5.2 8‐item sequences

We found similar results in the 8‐item condition. Using the same predictor variables of condition, touch number (for the second half of the list), and their interaction, we found that the best fitting model contained both of these effects as well as the interaction term (*χ* ^2^(1) = 11.42, *p* < .001). Overall, the second half of the center‐embedded sequences took longer to produce than cross‐serial sequences (β_condition_ = −851.5, *t*(86.1) = −3.49, *p* < .001; see Supplementary Materials for full model description and results including random effects) and there was again an effect of touch number, such that in the base condition (center‐embedded), item‐to‐item response time decreased for successive touches as each trial progressed (β_touch_ = −185.7, *t*(87.2) = −8.64, *p* < .001) We also found a significant interaction between condition and touch number such that the center‐embedded condition had a steeper decrease in item‐to‐item response times compared to the cross‐serial condition (see Fig. 6; β_condition*touch_ = 103.4, *t*(85.1)= 3.44, *p* < .001). This steep negative slope for item‐to‐item response times in the center‐embedded condition is a signature of an iterative search through a queue in which items are responded to as soon as they can be accessed (Thomas et al., 2003). These response time analyses suggest that participants use a queue to represent both center‐embedded and cross‐serial sequences and are inconsistent with the possibility that participants use different memory architectures (stacks for center‐embedded and queues for cross‐serial) when producing sequences allowed by their grammar. As was the case for the 6‐item lists, these effects held when just including participants who reached greater than 80% overall accuracy on these 8‐item arrays (see Table S14). This persisting RT signature suggests that participants who had induced the correct grammar deployed a queue memory architecture to generate the cross‐serial and center‐embedded sequences allowable by their learned grammar.

## 3 Computational models

A central premise of this paper is that it should be possible to infer the structure of the memory processes drawn upon to generate sequences consistent with the induced grammars. Specifically, if memory stores data about order like a stack, people should be overall faster and more accurate in generating center‐embedded relative to crossed‐serial sequences; conversely, if memory stores data like a queue, people should be overall faster and more accurate in generating crossed‐serial relative to center‐embedded sequences. In addition, if memory stores data like a queue, the decrease in item‐to‐item RTs over the second half of the list should be steeper in the center‐embedded condition. And indeed, the previous analyses have demonstrated the accuracy and item‐to‐item response time predictions from the hypothesis that participants are using queues to implement sequences with both cross‐serial and center‐embedded sequences are borne out.

Here, we develop two memory models corresponding to the two different possible memory architectures—a stack and a queue—and fit both models to the experimental data. The data we model here are those from the further training trials on 4‐, 6‐, and 8‐item arrays. After the five uncorrected test trials discussed above (Fig. 4), participants received additional training trials on the same novel arrays, where an error led to the end of the trial. There were 20 such trials at length 4 and 40 such trials at lengths 6 and 8. Here, we analyze data from both correct and incorrect sequences. No analyses from *incorrect sequences* during further training trials were included in the results presented so far, so these analyses provide new information that may converge with, or deviate from, the conclusions we have drawn so far.

The models allow us to investigate the sources of errors—whether they derive from an incorrect grammar or from difficulty in implementing the sequences specified by the grammar. That is, we explore whether the greater error rate in the center‐embedded condition is derived from greater difficulty learning the correct grammar, greater difficulty in producing the correct sequences licensed by that grammar, or some combination of both.

The grammars are implemented in programs that people may use to generate sequences in this experiment: one program that generates center‐embedded sequences and a program that generates crossed‐serial sequences. These programs get compiled into a list of push/pop operations from either a stack or a queue—the exact series of push and pop operations needed to pick each item to touch next in a center‐embedded versus crossed‐serial sequence depends on which memory architecture is used. The queue and stack architectures thus make distinct predictions about how quickly people will choose each item when generating center‐embedded and crossed‐serial sequences. Additionally, we included a program that generates sequences with blue items first and red items second, with no systematic order of the blue or red items. Because the further training trials stop immediately at the first incorrect touch, we did not attempt to differentiate between various other possible incorrect programs. This program does not rely on the use of either a stack or a queue, and thus, to the extent that participants were using this (incorrect) program to generate sequences, they would not distinguish them (see Fig. S4).

Finally, we also assume that the memory operations introduce the possibility of corruption (noise), which implies that the chance of making a mistake increases with the number of push/pop operations involved—so, for example, if the memory architecture is a queue, people should make more errors generating a center‐embedded sequence than a crossed‐serial sequence, since there are a greater number of push/pop operations involved and hence a greater possibility that memory will be corrupted.

As detailed in Supplementary Materials, we assume that participants generate a response as soon as they can identify the icon that should be selected. We model the RTs from one response to the next until there was an error, if there was an error. (This is unlike the data we reported above, where RTs were only included for completely correct sequences and only tested on the second half of the list.) We assume that there are multiple potential sources of error in generating sequences: people may be using the wrong program, implementing a program incorrectly due to a memory error, or simply not paying attention. We account for each of these possibilities in the model with independent parameters. It may be that participants do not immediately grasp how to pick items in the right order (e.g., they may think the rule is simply “pick blue items then pick red items,” without respect to order of the shapes). To account for this, we include a latent parameter representing the probability that participants are using the correct program on a given trial. This parameter is allowed to change over the course of training at each sequence length, following a Gompertz function (see Fig. S4). In addition to using the wrong program, another significant source of error may be misremembering the order of previously chosen items or even forgetting that an item has already been chosen. This is modeled by a different parameter, assumed to be constant for a given participant across the experiment, specifying how likely memory is to be corrupted each time an item is stored. We allow two types of corruption, “swaps” (reordering items in memory) and “deletions” (removing items from memory), which we assume for simplicity are equiprobable. Finally, we allow that on some proportion of trials, participants may not be paying attention or not trying, so they may be picking items at random. Additionally, there are parameters in the model to capture response times under both a stack and queue memory architecture—these are detailed in the Supplementary Materials.

### 3.1 Model comparison

We found maximum likelihood parameter estimates for each participant during the further training trials under both the queue‐ and stack‐based models. The likelihood was the product of the probability of each particular response (which item was pressed) and the response time to generate that response, given the set of model parameters. The probability of each response and response time was computed by simulating 100,000 possible responses from the model for each sampled set of parameters. We then computed the AIC difference between the queue and stack models, which was just dependent on the difference in likelihoods since they had the same number of free parameters. The details of model fitting can be found in Supplementary Materials.

**Fig. 7:**
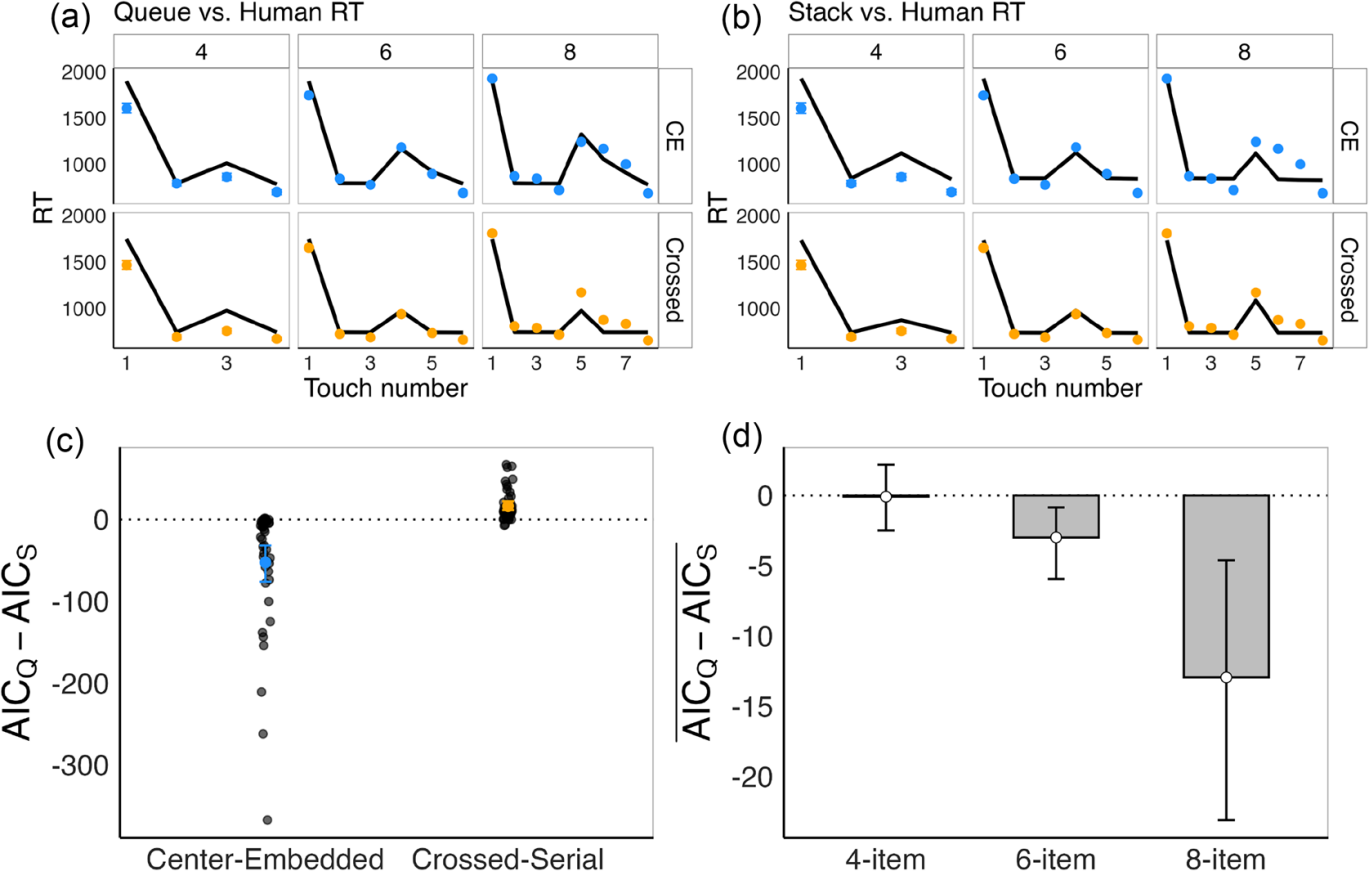
(a) Model predictions (black lines) and human data (colored points) for mean item‐to‐item response times as a function of the touch number within each sequence, in 4‐, 6‐, and 8‐item lists (panels, left‐to‐right). The queue model's predictions are on the left, and the stack model's predictions are shown on the right. The center‐embedded data is shown in blue on top, and the crossed‐serial data is shown in orange on the bottom. (b) The AIC difference of each subject under the queue and stack models in the center‐embedded and crossed‐serial conditions; lower values indicate a better fit of the queue model relative to the stack model. Colored dots show the average subject‐level AIC difference. (c) The overall mean AIC difference per subject between the queue and stack model in the 4‐, 6‐, and 8‐item lists collapsed across conditions.

Fig. 7a shows how the queue (left) and stack (right) models fit the aggregate item‐to‐item response‐time data in the center‐embedded (blue, top) and crossed (orange, bottom) condition. The mean model predictions are shown as black lines, and the human data are shown as colored points.

The differences between the two models are relatively subtle overall, but clear in the 8‐item center‐embedded condition. In that case, the stack model fails to capture the key downward‐sloping trend of the item‐to‐item response times in the second half of the list, whereas the queue model captures this trend closely. Fig. 7b shows the AIC difference between the queue and stack model for each subject in each condition, where lower values (more negative) indicate a better fit of the queue model over the stack model. The group‐level data from each condition point in contradictory directions, with a slightly better fit of the stack model in the crossed‐serial condition (Group Level ΔAIC = 791) and a significantly better fit of the queue model in the center‐embedded condition (Group Level ΔAIC = 2250).

Why does the stack model provide a slightly better fit in the cross‐serial condition than in the center‐embedded condition? No theories posit that stacks are used for cross‐serial sequences but not center‐embedded ones. The sole aspect of the data that drives this effect is that stack architecture predicts that participants will take increasingly longer to respond to the first item of the second half of the list as list length increases, because of the additional push/pop operations needed as list length increases. And indeed, this is observed (see Fig. 7a, and note the good fit of the stack model to the first item in the second half of the list in the crossed serial condition). However, as Fig. 7a shows, this pattern of increasing position‐specific response time for the first item in the second half of the list as list length increases is also observed in the center‐embedded condition, where it is not predicted on the stack model. Moreover, this increasing response time as list length increases is also observed on the very first touches of the blue items in both conditions, where it is not predicted on either model.

This pattern is highly systematic but does not provide clear evidence in favor of either memory architecture. It is plausible that upon seeing the arrays for the first time, participants scan the blue items to decide which order to touch them in, and this takes more time as the lists increase from 4 to 6 to 8 items. Similarly, before switching to the red items, they again scan all the red items before beginning, and this also takes more time as the lists increase from 4 to 6 to 8 items. We conclude that our data provide no evidence for the deployment of stacks in this grammar induction/sequence production task.

### 3.2 Analyzing the sources of error

Given the evidence that participants are using queues to generate the center‐embedded sequences and no clear evidence for the stack model in either sequence type, we now use the queue model alone to better understand why participants made errors—and specifically why they made them at different rates in the two conditions. We assumed that there were three potential reasons participants might make an error: (1) they were using a program that failed to generate sequences with the correct grammar beyond blue before red; (2) they intended to generate a correct sequence but misremembered which items they had already chosen or the order in which they were chosen; or (3) they were not paying attention or pressing randomly. Each of these potential sources of error would generate different error patterns: the first would create errors in the second half of the list, particularly toward the beginning of the second half of the list; the second would generate patterns that are close to a correct sequence but have an error, particularly likely toward the end of the sequence; and the third would generate an equal proportion of errors across the entire sequence. We included parameters for each of these possibilities in the model, allowing us to determine the relative contribution of each source of error (see Supplementary Materials).

**Fig. 8:**
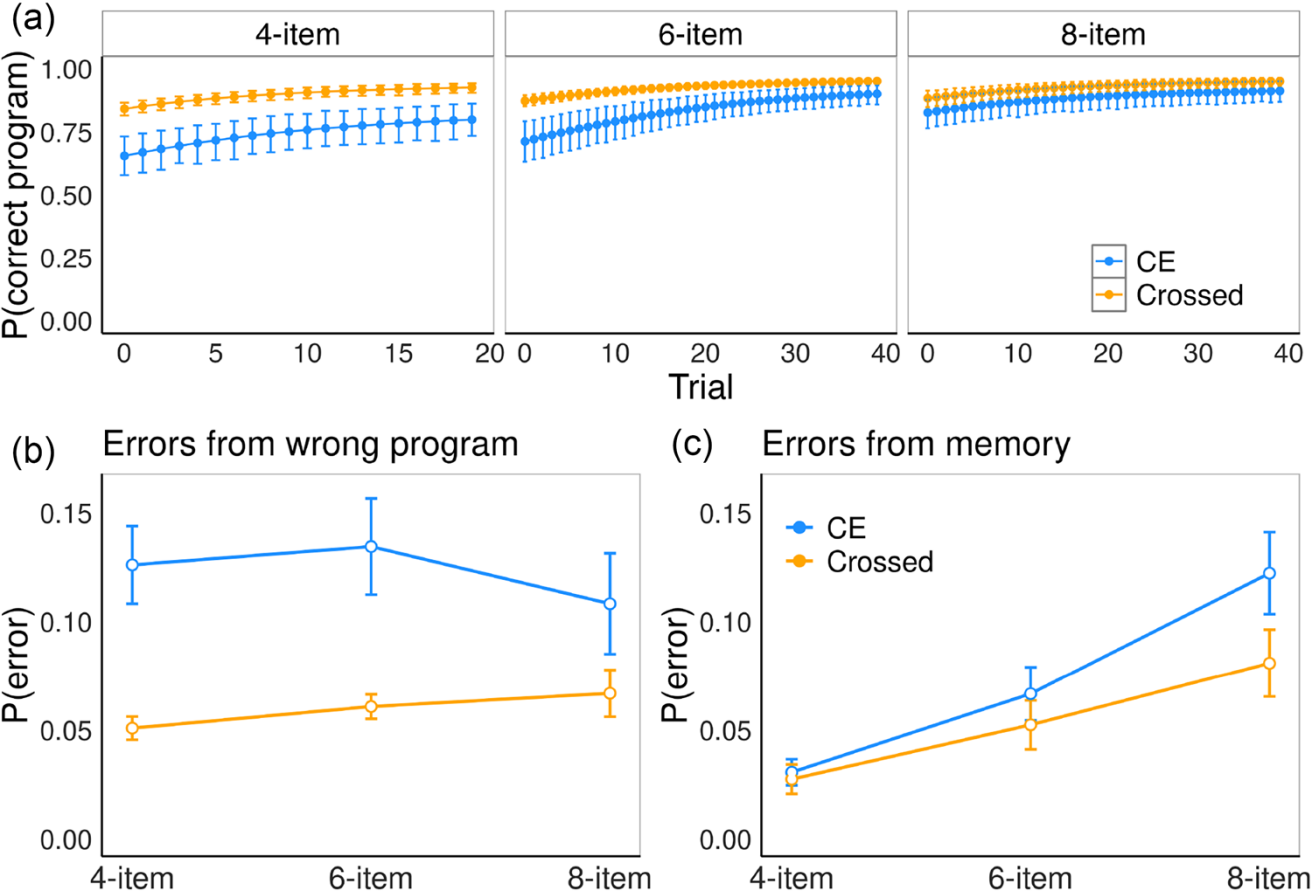
(a) The inferred probability that participants are using the correct program over the course of training trials of each sequence length, in both the center‐embedded (blue) and crossed‐serial (orange) conditions. (b) The average probability that participants make an error from using the wrong program across 4‐, 6‐, and 8‐item lists. (c) The probability that participants make an error from misremembering which items they already chose or the order in which they chose them.

We assumed that participants may not have learned the correct program before the beginning of each new sequence length, but may learn it over the course of the training trials. We, therefore, fit participants’ probability of using a correct program as a function of trial number under a Gompertz curve, which allows us to fit a learning asymptote, slope, and intercept. Fig. 8a shows the inferred average probability of using the correct program over the course of training trials in each sequence length in both conditions. See Supplementary Materials for modeling details. The difference between the two conditions is substantial for 4‐ and 6‐item sequences, especially at the beginning of the set of trials, but small for 8‐item sequences and at the end of the 4‐ and 6‐item training trials as well. This indicates that learning the center‐embedded grammar is substantially harder than learning to crossed‐serial grammar. Overall, the inferred rate of errors from the incorrect program was twice as great in the center‐embedded condition (0.12, CI = [0.10, 0.15]) than in the crossed‐serial condition (0.06, CI = [0.05, 0.07]).

We can compare the rates at which errors were caused by difficulty learning the correct program relative to difficulty implementing that program. Fig. 8b shows the rate at which errors were caused by using the wrong program, and Fig. 8c shows the rate at which errors were caused by misremembering which items were previously chosen or their order. Errors caused by using the wrong program were more common across all sequence lengths in the center‐embedded condition relative to the crossed condition, and were more common earlier in learning (i.e., 4‐ and 6‐item sequences). This analysis underlines what we have said before; learning a sequence length independent rule equivalent to “put the red items in reverse order to the blue items” was difficult, and not achieved by every participant, even by the end of training on the 8‐item lists. There were even a small percentage of errors due to wrong program in the cross‐serial condition (Fig. 8b). In contrast, the effect of memory noise only causes substantially more errors in the center‐embedded condition relative to the crossed‐serial condition in the 8‐item sequences, with a rate of 0.12 (CI = [0.10, 0.14]) memory errors in the center‐embedded condition and a rate of 0.08 (CI = [0.07, 0.10]) memory errors in the crossed‐serial condition. As would be intuitively expected, the rate of errors caused by memory noise grows with the number of items displayed—but it does so more quickly in the center‐embedded condition, as predicted by the queue model but not the stack model (Fig. 8c and Fig. S5).

## 4 General discussion

There were five main results from this grammar induction study. First, this task was not trivial for human adults. Learning of the grammars was not complete even by the end of the study. Second, sequencing performance was well above chance on 6‐ and 8‐item *novel* test arrays. This establishes that at least some participants had abstracted grammars that generated full center‐embedded and cross‐serial sequences and generalized to more levels of embedding than in their training. Third, participants in the center‐embedded condition required more training to learn a grammar that generated allowable sequences than did those in the cross‐serial condition. This difference was seen from the very beginning of training, but especially in the generalization from one level to embedding to two. This result replicates other studies in finding that center‐embedded grammars are harder to learn and/or implement than closely matched cross‐serial grammars (Bach et al., 1986; De Vries et al., 2012; Öttl et al., 2015; Uddén et al., 2009; Uddén et al., 2012). Fourth, participants in the center‐embedded condition had more difficulty implementing the procedure that generates such sequences once learned than did those in the cross‐serial condition, as shown by longer RTs between touches in the second half of the list in the center‐embedded condition. Fifth, over the second half of the list, the item‐to‐item RTs decreased significantly more in the center‐embedded condition than in the cross‐serial condition, which is a signature of a queue‐like memory architecture.

### 4.1 Memory architectures for lists

It is often assumed that humans use push‐down stacks to represent center‐embedded and cross‐serial sequences (Fitch & Hauser, 2004; Jiang et al., 2018; Joshi, 1990; Joshi, Shanker, & Weir, 1991; Kinsella, 2010; Malassis, Dehaene, & Fagot, 2020; Rodriguez & Granger, 2016). The finding that cross‐serial grammars are easier to learn (and it is easier to produce corresponding sequences) is inconsistent with the assumption that push‐down stacks are the basis of representing both types of grammars, because cross‐serial sequences would require two stacks (and additional processing), relative to center‐embedded sequences, which would require only one (see Fig. 1).

However, the relative difficulty in learning/execution is consistent with the possibility that push‐down stacks are used when generating center‐embedded sequences, some other representation of sequences is used for cross‐serial sequences, *and* that push‐down stacks make greater information processing demands than the alternatives. To evaluate this hypothesis, we examined item‐to‐item RTs as well as error rates and compared a stack architecture with an alternative: a queue. We found strong evidence that queues were used to represent and execute center‐embedded and sequences in this study. As predicted by the queue model, participants in the center‐embedded conditions showed steeper linearly decreasing item‐to‐item response times for the second half of the sequence than did those in the cross‐serial conditions. This reflects the fact that participants in the center‐embedded condition had to iteratively step through the queue memory architecture to find the next item in the sequence. A push‐down stack memory architecture would predict flat RTs as you can just pop each successive item off the stack (Silva et al., 2022; Uddén et al., 2012). A whole list memory architecture in which all items can be accessed at once would also predict flat RTs, as the items on the list could be accessed from the beginning or the end (McElree & Dosher, 1993). Thus, the observed response time findings, along with the robust evidence that center‐embedded grammars are harder to learn and generate than cross‐serial ones, point to a queue‐based memory architecture being used to create and evaluate center‐embedded and cross‐serial sequences in the A^n^B^n^ indexed grammar learning tasks.

These conclusions were further explored in a comparison of two computational models, one based on stacks as its memory architecture and one based on queues as its memory architecture, in the fit to the observed data in the generation of both cross‐serial and center‐embedded sequences. Overall, the queue model fit the data better. Accordingly, we used the queue model to separate sources of error into two categories: (1) from having failed to learn the correct sequence, and (2) from memory errors due to the difficulty implementing the process that produces the correct sequences. The modeling results found that both sources of error were greater in the center‐embedded condition. Furthermore, separating these sources of error confirmed that errors due to *learning* the correct center‐embedded grammar were greater at the beginning of the experiment (i.e., more evident on the 4‐ and 6‐item novel lists than on the 8‐item novel list), whereas, not surprisingly, *memory errors* increased with the length of the list.

### 4.2 Did participants learn unbounded grammars?

We tested sequence lengths of up to 8 items. Thus, we cannot conclude that the grammars induced were truly unbounded. Participants could have learned either a bounded grammar (A^n^B^n^, 0 ≤ n ≤ 4) or an unbounded grammar (A^n^B^n^, 0 ≤ n). However, to induce the bounded version of this grammar, participants would have needed to acquire a grammar (e.g., “A^n^B^n^, 0 ≤ n ≤ 4”) at the 6‐item stage in order for it to be used to generalize to the never‐before‐seen 8‐item length. Inducing this type of bounded grammar for an unobserved number of embeddings seems highly unlikely (McCoy et al., 2021). Although there is no way of testing for unbounded grammar learning in the sequencing task used here, future work could ask subjects to specify the grammar learned, that is, to describe the procedure they were using to generate sequences, which could provide further evidence of the type of grammar induced.

### 4.3 Questions for further research

These data raise several questions for further research and discussion. The first question is why center‐embedded grammars were harder to *learn* than were cross‐serial grammars in this sequencing task. The conclusion that queues are used to represent both cross‐serial and center‐embedded sequences makes sense of why center‐embedded sequences were harder to *produce online*, generating more errors in sequencing and the distinctive item‐to‐item RT profile of iterative stepping through a queue in the second half of the list. But why was the center‐embedded grammar harder to learn?

To learn what the grammar is, a participant must *notice* that at least one training sequence had a center‐embedded structure in order to *hypothesize* that the sequences might always satisfy a center‐embedded grammar. Both center‐embedded sequences and cross‐serial sequences require representing the order of touches of the blue items, and in these studies, these are represented as a queue. Thus, the “same queue order” hypothesis is immediately available as a hypothesis for the order of the red shapes. To even notice the “reverse queue” order for the red shapes, one must iteratively step through the queue. That is, it is quite possible that the greater processing difficulty of generating center‐embedded sequences from queue representations makes them harder to learn, as well as harder to generate online even once learned.

Accepting this argument has major implications for the original motivation for the project that led to comparing the ease of learning cross‐serial and center‐embedded grammars and producing these sequences: namely, to explore whether formal grammar complexity (in terms of the Chomsky−Shutzenberger hierarchy) has implications for the *mental representations* of these grammars. We find no evidence that formal complexity predicts relative processing difficulty. Complexity matters to ease of learning, but it is processing complexity, not formal complexity, that does so.

Another important question for further research concerns the capacities of nonhuman animals and young children on the present grammar induction task. Ferrigno et al. (2020) showed that both rhesus macaques and 3‐ to 5‐year‐olds could learn the two initial training sequences in the center‐embedded condition (the cross‐serial condition was not tested) and generalize what they had learned to the novel combination test trials. The monkeys were also tested on completely novel 4‐item test arrays, and after enough experience, successfully induced a grammar that generalized to novel sequences. Subsequent work with 3‐ to 4‐year‐olds added a cross‐serial condition and tested generalization to novel test arrays (Ferrigno & Carey, 2020). This work found that the center‐embedded grammars were harder to learn and the corresponding sequences were harder to process than cross‐serial ones, as with adults. Also, as with adults, there was successful generalization to novel 4‐item arrays in both cases. What is not known is whether the children had learned only a bounded grammar that generates only the correct 4‐item sequences (i.e., a fixed‐length regular grammar). Such a grammar would not generalize to novel 6‐ and 8‐item sequences.

Recent work by Liao et al. (2022) found that crows could represent 6‐item center‐embedded sequences with some training, but they did not test for spontaneous generalization from 4‐ to 6‐items. A high priority is to establish whether cross‐serial and center‐embedded grammars that generalize to additional levels of embedding can be learned on the basis of the 4‐item training by nonhuman animals and young children. Ferrigno (2018) provided some suggestive evidence that this is within the competence of nonhuman primates: The single monkey tested showed some generalized from training on 4‐item sequences to a novel 6‐item sequence. If further work finds failure in such generalization under the training regimes of that study, which were similar to those deployed here, the next question would be whether a grammar that generates 6‐item sequences *can* be learned with further training on 6‐item sequences by these populations, and whether, as was the case in this study, the animals can generalize to a new level of embedding (e.g., 8‐item sequences).

Another question raised by this research concerns the nature of the learning mechanism that abstracts these grammars. Computational modeling approaches might provide insights into how different grammars are learned. Note, the model we report here is not a model for *how* the grammars are learned. Rather, it models *when* some grammar sufficient to generate grammatical sequences has been learned, and the sources of errors as a function both of not having learned a relevant grammar and difficulty executing the process that generates correct sequences.

A recent paper by Yang and Piantadosi (2022) takes on the learning problem, albeit not with the goal of elucidating the learning processes in experiments such as these. Rather, its goal is to challenge arguments that grammars of natural language are not learnable in the absence of innate domain‐specific constraints. Yang and Piantadosi tested 82 different grammars, including regular, context‐free, and context‐sensitive grammars, many of them vastly more complicated than those studied here, as well as fragments of natural language grammars. They showed that almost all are learnable from small amounts of positive data alone. The model included logical and set manipulation primitives, along with a few representational/computational primitives related to representations of lists. These list primitives included *list*, or *string*, and a few functions on lists including adding a new character on the end of the list, identifying the first character of the list, identifying the rest of the list, appending or joining lists *X* and *Y*. Interestingly, there is no predefined function that identifies the last character in the string, although it would be easy to build a procedure for doing so from the primitives of the system. Thus, the above functions on lists have the properties of functions on queues, not stacks. However, there is one primitive function that makes the primitive list structures unlike either stacks or queues, namely, *insert (X*, *Y)*, which inserts list *X* into the middle of list *Y*. This requires a representation that can access a whole list at once.

Although the Yang and Piantadosi (2022) model was not intended to capture the learning process at an algorithmic level, it opens a dialog about the actual learning mechanisms that induce grammars from encountering strings that satisfy the grammar, and, like the present study, calls for further research on the nature of list representations and the list manipulation functions animals and people actually deploy.

### 4.4 Relations between the grammars in the present study and those in natural language

As previously mentioned, center‐embedded structures in German are harder to *process* than the comparable cross‐serial structures in Dutch (Bach et al., 1986). Whether center‐embedded structures in German are harder to *learn* than are the comparable cross‐serial constructions in Dutch in the course of natural language acquisition is not yet known. Comparative study of the acquisition of these structures by Dutch‐learning and German‐learning children could show whether the parallels between the artificial language and linguistic structures regarding processing difficulty extend to difficulty in learning.

Whatever the outcome, there are many reasons to doubt that the present results bear directly on the acquisition or implementation of center‐embedded structures or cross‐serial structures in natural language. There are many important differences between these structures in natural language from those implemented in the artificial grammar tasks we have discussed in this paper. One difference is that in natural language, the A B *indexed pairs* are related to each other by predication, modification, quantification, or other semantic relations, whereas in the artificial language studies, the pairing is arbitrary or is based on similarity. Another difference is that in natural language, each clause not only contains another clause but is modified by it. These differences limit the conclusions that can be made from the present results as to whether center‐embedded grammars in natural language should be harder to learn or harder to process than cross‐serial grammars.

One potential avenue for future research would be to make the base pairs relationally related, such as dog/bone, cat/milk, mouse/cheese, and study how hard it is to learn and implement the grammars. Once learned, these grammars could be sources of learning novel animal/food pairings from the positions in whole novel sequences. We are engaged in such studies. A similar line of work has shown interactions between syntax and semantics when learning artificial center‐embedded grammars. Poletiek, Monaghan, van de Velde, and Bocanegra (2021) tested participants in an artificial center‐embedded language task that included scenes illustrating the intended meaning. Participants not only learned the syntax of the artificial grammar but were also able to interpret the meaning of novel sentences in the artificial language.

Another likely difference between the grammars of natural language and those learned in the present studies is that natural language grammar is implicitly represented. It takes linguistics to articulate the strong generativity of natural language (e.g., a set of structural descriptions). In contrast, even though subjects were only tested on their ability to generate sequences that fit the grammars (weak generativity), it is very likely that participants represented the grammars in the present study as explicit rules, such as “blue then red, make the order of shapes of the reds the same (or reversed) of the blues.” Follow‐up studies should test whether participants have an explicit formulation (i.e., strong generativity) of the grammar they are following *at any given point* in the study. Notice, many participants had not formulated an explicit grammar such as the above, for if they had, they would be at ceiling on the task. None of the sequences had a list length above 4 for the blue items, which is well within the limits of adult limits on representing an ordered list in working memory. Follow‐up studies should also explore the important question of whether the results would extend to a task that encourages implicit learning of grammars that generate cross‐serial and center‐embedded sequences with 1, 2, and 3 levels of embedding (Reber, Walkenfeld, & Hernstadt, 1991; Seger, 1994).

Imaging (fMRI) studies provide evidence that the language network is distinct from the multiple‐demand network (e.g., Blank, Kanwisher, & Fedorenko, 2014). If a grammar is explicitly formulated in the current grammar induction and sequencing task, which we expect is the case, then participants would likely recruit the multiple‐demand network rather than the language network. Follow‐up studies using fMRI could establish this.

Finally, while the response time data in the present experiment showed evidence of queue representations of the ordered lists, there are empirical reasons to doubt that this is so in natural language comprehension. Perhaps most directly, McElree, Foraker, and Dyer (2003) studied the nature of the memory representations deployed in understanding gap/filler sentences with 0, 1, or 2 embedded clauses between the word that was the subject or object of a verb and the verb itself. Using Speed Accuracy Tradeoff methods, they could assess whether lists held in queues were being searched through. Instead, they found the signatures of content addressability, just as was the case in the working memory experiments mentioned above (e.g., McElree & Dosher, 1993). That is, the number of interpolated clauses affected overall accuracy, but had no effect on speed of processing, the latter being the signature of a search process through a memory file of a list.

### 4.5 Why are center‐embedded structures difficult in natural language processing?

There is a large literature on why center‐embedded structures are more difficult to understand than are tail‐embedded or cross‐serial structures in natural language (e.g., Christiansen & Chater; 1999; Christiansen & MacDonald, 2009; Coopmans, De Hoop, Kaushik, Hagoort, & Martin, 2022; Gibson, 1998; Joshi, 1990; Lakretz et al., 2021). Reviewing this work is outside the scope of this paper, as the goal of this research here was not to contribute to this literature. With the exception of Joshi (1990), this literature does not concern itself with the memory architectures that represent linear order; Joshi assumes a stack architecture. In all cases, including Joshi's work, this literature appeals to the details of natural language and the relations between the grammars and parsing processes; that is, it explicitly draws on the aspects of linguistic sequence structures that differentiate them from those in the artificial grammar studies addressed here.

In sum, there are many reasons to expect that the processes that compute the nested, center‐embedded, sentences in natural language differ deeply and fundamentally from those of the simple artificial grammars deployed here. Nonetheless, this does not detract from the further point that, however, people compute the structure of a sentence during comprehension, they must have derived that structure from information carried in the linear order of the words they heard, at least in part. Similarly, they must also create representations in an ordered list during production. The parallel results in the current artificial grammar study and in natural language are also consistent with a common explanation—that queues may sometimes be used to represent linear orders within natural language at some point of the processing. To explore this hypothesis, one must test alternative memory architectures and the actual retrieval processes that are deployed in natural language.

## 5 Conclusion

To our knowledge, the present study is the first to use accuracy and item‐to‐item response times to test how humans represent indexed A^n^B^n^ grammars. We test if the memory structures humans use to represent these complex grammars are stacks (representations of string order that can only be accessed from the end) or queues (representations of string order that can only be accessed from the beginning). Drawing on the literature on forward and backward list recall, we found no evidence that the mind deploys stacks in these grammar induction paradigms, and positive evidence that participants deployed queues in the learning of both simple context‐free and simple context‐sensitive grammars. We also found evidence that participants deployed queues in the production of sequences that satisfied both context‐free and context‐sensitive grammars.

## Competing interests

The authors declare no competing interests.

## Data and code availability

All data and code for the main analyses are available here: https://github.com/Sferrigno/Do‐humans‐use‐push‐down‐stacks‐when‐learning‐or‐producing‐center‐embedded‐structures. Code for the Bayesian Modeling section can be found here: https://github.com/samcheyette/stacks_and_queues

## Supporting information



Supplemental Information

## Notes

1 Chomsky (1965, pp. 62) introduced the distinction between weak and strong generativity in the following passage: “It may be useful to make one additional methodological observation in connection with the topics discussed in the last few sections. Given a descriptive theory of language structure, we can distinguish its ***weak generative capacity*** from its ***strong generative capacity*** in the following way. Let us say that a grammar ***weakly generates*** a set of sentences and that it ***strongly generates a*** set of structural descriptions (recall that each structural description uniquely specifies a sentence, but not necessarily conversely).”

2 This animal work was couched in terms of the question of whether the capacity for recursion is human unique. We do not frame our work in terms of “recursion,” for this term is ambiguous between two fundamentally different senses: “recursive functions–procedures that call themselves” and “recursive structures–sequences of spatial structures in which a structure is embedded within one of the same kind i.e., center‐embedded structures.” As Lobina (2011) has extensively documented, there is no direct relations between recursive operations and recursive structures. Any structure, center‐embedded or not, can be generated either by recursive operations or nonrecursive operations. Nonetheless, we believe that the question of whether nonhuman animals can learn grammars, generative procedures that create novel sequences with shared structures, is important in the context of what foundational capacities may be human unique.

3 Examples of cross‐serial and center‐embedded structures from Bach et al. (1986). Both sentences express that the men have Hans teach the horses to feed, but note in the glosses, the difference between the Dutch cross‐serial sequence and the German center‐embedded sequence.(1) Dutch: De mannen hebben Hans de paarden leren voeren. (Cross‐serial)*The men have Hans the horses teach feed*.(2) German: Die Manner haben Hans die Pferde futtern lehren. (Center‐embedded)*The men have Hans the horses feed teach*.

4 Although the description of a queue memory architecture for center‐embedded sequences includes stepping through the queue multiple times. This process can also be described in terms of push/pop operations. Due to the nature of a queue, when you pop items off a queue and onto a second queue, the order remains the same (in contrast to a stack which reverses the order if items are moved from one stack to another). Importantly, these two descriptions are functionally equivalent. The number of operations (i.e., steps through a queue or push/pops) make the same response time predictions regardless of whether they are steps through a single queue or if items are pushed onto a second queue.

5 Uddén et al. (2012) found partial evidence for a stack‐like representation of a cross‐serial string. In the cross‐serial condition, participants were more likely to judge agrammatical strings as grammatical when the violation was in the first position of the second half of the list compared to other positions. This is predicted by a stack architecture because at this point, you would need to pop (and then push) each item off the original stack and onto a new one at this point (see Fig. 2a, stack architecture: cross‐serial string). However, the stack model also predicts that the first position violations in the cross‐serial condition should be less noticeable than any violations in the center‐embedded structure (where no additional popping or pushing is needed), but they found no evidence of this.
