# Supplementary material for: Do Humans Use Push‐Down Stacks When Learning or Producing Center‐Embedded Sequences?
Source: Cogn Sci. 2025 Sep 15;49(9):e70112. doi: 10.1111/cogs.70112 (PMC12435407; doi:10.1111/cogs.70112)
Supplement: Supplementary file 1 — Supplemental Information [file COGS-49-e70112-s001.pdf]

Supplemental Information

Exclusion Criteria

Initial Training Trials – Phase 1

To find and remove outliers from the training data, we plotted the number of trials to criterion using a box plot and saw that there were multiple outliers. To account for this, we removed the values of data points which lied beyond the extremes of the whiskers using the base R function "boxplot.stats out". There was a total of 12 outliers that were removed from the trials to criterion analyses, 5 in the center-embedded condition and 7 in the cross-serial condition.

Generalization Trials – Phase 2, novel combination of trained items

Any subject who touches the same picture >= 2 times on 50% more of the non-differentially reinforced training trials was excluded from that list length because this would indicate the subject didn't understand the task or was purposefully responding incorrectly. There were none of these Phase 2.

Individual no feedback generalization trials were be excluded if subjects touch the same image more than two times. There was one trial that was excluded for this reason in the center-embedded condition.

Generalization Trials – Phase 3, test trials with completely novel stimuli

Any subject who touches the same picture >= 2 times on 50% more of the non-differentially reinforced training trials was excluded from that list length because this would indicate the subject didn't understand the task or was purposefully responding incorrectly (4-item: center-embedded n = 1; 6-item: center-embedded n = 1, cross-serial n = 2; 8-item: center-embedded n = 5, cross-serial n = 1)

Individual no feedback generalization or transfer trials were be excluded if subjects touch the same image more than two times. There were 0 of these trials in the 4-item array size, 2 in the 6-item array size, and 2 in the 8-item array size.

Further Training Trials

Subjects who had an accuracy of > 2 SD from the mean of all subjects within the same condition were excluded from analyses (4-item: center-embedded n = 3, cross-serial n = 4; 6-item: center-embedded n = 3, cross-serial n = 3, 8-item: n = 5, cross-serial n = 4). The Bayesian analysis also used this exclusion criterion and did not exclude any specific trials based on response time.

For the response time analyses, we only include trials in which the entire sequence follows the correct pattern (either center-embedded or cross-serial depending on the condition). Any trial that has a total response time that is longer than >2 SD of the mean response time for all subjects in the same condition/list length were be excluded from the RT analyses. There was between 2% - 4% of trials in each condition that fell in this category (6-item center-embedded trials = 57, cross-serial trials = 74; 8-item: center-embedded trials = 55, cross-serial trials = 74). Additionally, Individual touches within a trial were excluded from the response time analysis if they were > 2 SD away from their own mean response times within trials of the same type. There were between 5%-10% of touches that fell in this category (6-item: center-embedded touches = 776, cross-serial touches = 1,123; 8-item: center-embedded touches = 789, cross-serial touches = 944).

Results

Full Regression Models and Results

Phase 1: 4-item array training

We used a mixed effects linear regression to quantify the effects of array order (first or second training array) and condition (center-embedded or cross-serial) on the number of trials to criterion (see Table S1). The inclusion of array order as a random effects term was a singular fit and was removed. We included a random effects term of participant to handle variability that arises from individual subjects.

Table S1 - Phase 1 - Trials to Criterion Results

	Trials to Criterion		
Predictors	*Estimates*	*CI*	*p*
(Intercept)	14.11	12.70 – 15.52	**<0.001**
Array Order [TrainingList2]	-8.06	-10.01 – -6.10	**<0.001**
**Random Effects**			
σ^2	43.07		
$\tau_{00\ participant}$	1.78		
ICC	0.04		
$N_{participant}$	88		
Observations	176		
Marginal R^2 / Conditional R^2	0.267 / 0.296		

Table S1: Fixed and Random effects from the linear regression predicting Trials to Criterion from the best fitting model which included only training array order.

Phase 1: 4-item array training

We used a mixed effects logistic regression to quantify the effects of condition (center-embedded or cross-serial) on the number of correct generalizations (see Table S2). The inclusion of condition by participant as a random effects term was a singular fit and was removed. We included a random effects term of participant to handle variability that arises from individual subjects. We found that the best fitting model included condition ($\chi^2(1) = 5.33$, $p = 0.021$, compared to the null model).

Table S2 - Phase 2 - Novel combination of base pairs

	Correct Generalizations		
Predictors	*Odds Ratios*	*CI*	*p*
(Intercept)	16.52	5.15 – 52.97	**<0.001**
Condition [Crossed]	5.42	1.30 – 22.57	**0.020**
**Random Effects**			

σ^2	3.29
τ_{00} participant	8.66
ICC	0.72
N participant	100
Observations	999
Marginal R^2 / Conditional R^2	0.056 / 0.740

Table S2: Fixed and Random effects from the logistic regression predicting correct generalizations from condition.

Phase 3: Completely novel 4-item, 6-item, & 8-item arrays

We used a mixed effects regression analysis to quantify the effects of condition (center-embedded or cross-serial), array size (4-, 6-, or 8-item arrays), and their interaction on the probability of producing the correct generalization (see Table S3). We included the random effects terms including by subject random intercepts for condition and array size. We found that the best fitting model included fixed effects for condition, array size, and their interaction. Inclusion of a by subject random effects term of interaction did not improve the model fit. We used the "bobyqa" optimizer to handle any convergence issues.

Table S3: Phase 3 - Logistic Regression Results

	Correct Generalizations		
Predictors	*Odds Ratios*	*CI*	*p*
(Intercept)	7.66	3.45 – 16.98	**<0.001**
Condition [Crossed]	2.66	0.71 – 9.92	0.146
Array Size [genList6]	0.06	0.03 – 0.17	**<0.001**
Array Size [genList8]	0.17	0.06 – 0.47	**0.001**
Condition [Crossed] * Array Size [genList6]	4.88	1.09 – 21.85	**0.038**
Condition [Crossed] * Array Size [genList8]	1.05	0.23 – 4.76	0.945
**Random Effects**			
σ^2	3.29		
τ_{00} participant	3.37		
τ_{11} participant.ConditionCrossed	6.43		
τ_{11} participant.ArraySizegenList6	3.68		
τ_{11} participant.ArraySizegenList8	4.65		
ρ_{01}	-0.62		
	0.58		
	0.37		
ICC	0.64		

N participant	99
Observations	1446
Marginal R^2 / Conditional R^2	0.141 / 0.690

Table S3: Fixed and Random effects from the logistic regression predicting correct generalizations from condition and array size.

To test for specific differences between condition (collapsed across array size), array sizes (collapsed across condition), and differences between 4-, 6-, and 8-item arrays in each condition we ran a series of pairwise comparisons and used the tukey method to account for multiple comparisons (see Table S4-6).

Table S4 - Pairwise comparison of condition averaged over array size

contrast	estimate	SE	df	z.ratio	p.value
Center - Crossed	-1.52	0.512	Inf	-2.972	0.003

Results are averaged over the levels of: round
Results are given on the log odds ratio (not the response) scale.

Table S4. Pairwise comparison between conditions, averaged over of array size. There was a significant difference in condition with cross-serial having a higher proportion correct.

Table S5 - Pairwise comparison of array size averaged over condition

contrast	estimate	SE	df	z.ratio	p.value
genList4 - genList6	1.945	0.378	Inf	5.151	<.0001
genList4 - genList8	1.736	0.38	Inf	4.566	<.0001
genList6 - genList8	-0.209	0.303	Inf	-0.688	0.7702

Results are averaged over the levels of: Condition
Results are given on the log odds ratio (not the response) scale.
P value adjustment: tukey method for comparing a family of 3 estimates

Table S5. Pairwise comparison of array size, averaged over condition. There was a significant difference between the 4- and 6-item array size, and the 4- and 8-item array size, but no difference between the 6- and 8-item array sizes when the results were averaged across conditions.

Table S6 - Pairwise comparison of array size and condition

contrast	estimate	SE	df	z.ratio	p.value
Center genList4 - Crossed genList4	-0.977	0.673	Inf	-1.452	0.6948
Center genList4 - Center genList6	2.737	0.485	Inf	5.648	<.0001
Center genList4 - Crossed genList6	0.175	0.574	Inf	0.306	0.9996
Center genList4 - Center genList8	1.762	0.51	Inf	3.455	0.0073
Center genList4 - Crossed genList8	0.733	0.5	Inf	1.465	0.6865
Crossed genList4 - Center genList6	3.714	0.767	Inf	4.841	<.0001
Crossed genList4 - Crossed genList6	1.152	0.586	Inf	1.968	0.3611
Crossed genList4 - Center genList8	2.739	0.762	Inf	3.594	0.0044
Crossed genList4 - Crossed genList8	1.709	0.57	Inf	3.001	0.0322
Center genList6 - Crossed genList6	-2.562	0.688	Inf	-3.725	0.0027
Center genList6 - Center genList8	-0.974	0.435	Inf	-2.239	0.2197
Center genList6 - Crossed genList8	-2.004	0.619	Inf	-3.24	0.0152
Crossed genList6 - Center genList8	1.587	0.68	Inf	2.333	0.1807
Crossed genList6 - Crossed genList8	0.557	0.436	Inf	1.277	0.7978
Center genList8 - Crossed genList8	-1.03	0.621	Inf	-1.66	0.5587

Results are given on the log odds ratio (not the response) scale.
P value adjustment: tukey method for comparing a family of 6 estimates

Table S6. Pairwise comparison of array size and condition. There were a number of significant differences, but notably an improvement in performance from the center-embedded 6-item to the center-embedded 8-item with no such difference in the cross-serial.

Response time analyses (correct sequences in the further training trials: 6-, and 8-item arrays)

We used a mixed effects linear regression analysis to quantify the effects of item number, condition (center-embedded or cross-serial), and their interaction on the probability of producing the amount of time for each touch (see Table S7). We first ran the full model with the maximal random effects structure including by subject random effects terms of touch, condition, and their interaction. We used the "bobyqa" optimizer to handle any convergence issues. This full model did not converge, so random effects terms were removed until the model converged. The model that converged included a by subject random effects term of "1 + Touch | Participant" to capture differences in the effects of touch number on individual subjects. We ran this on both the 6- and 8-item array length.

Table S7: Response time by item number

	6-item Array			8-item Array		
Predictors	*Estimates*	*CI*	*p*	*Estimates*	*CI*	*p*
(Intercept)	1493.54	1273.71 – 1713.36	**<0.001**	2188.48	1846.55 – 2530.42	**<0.001**
Condition [Crossed]	-640.82	-949.16 – -332.49	**<0.001**	-851.35	-1329.45 – -373.24	**<0.001**
Touch	-136.71	-173.98 – -99.43	**<0.001**	-185.74	-227.90 – -143.59	**<0.001**
Condition [Crossed] * Touch	100.44	48.22 – 152.66	**<0.001**	103.37	44.52 – 162.22	**0.001**

Random Effects

σ^2	48338.50	115962.98
τ_{00}	479859.37 participant	1242690.62 participant
τ_{11}	13357.93 participant.Touch	18297.09 participant.Touch
ρ_{01}	-0.96 participant	-0.98 participant
ICC	0.46	0.39
N	94 participant	91 participant
Observations	5913	8166
Marginal R^2 / Conditional R^2	0.046 / 0.480	0.082 / 0.440

Table S7: Fixed and Random effects from the linear regression predicting Response Time using Condition, Touch Number, and their interaction for both the 6- and 8-item array lengths. We used the touch numbers from the second half of the list, not including the first touch in the second half of the list because these response times could reflect the time to start producing the second half of the list (e.g., the time to switch from pushing items onto a stack to popping them, or the time to begin searching through a queue).

Preregistered analyses not included in main text

There were three analyses that were included in the preregistration that are not reported in the main text because they were redundant with the Bayesian Analysis section (further training trial accuracy and accuracy by position) or included within a larger regression (overall response time results). These three analyses are presented below.

Further Training Trials

To test if accuracy in each of the conditions and array sizes were above chance, we ran a series of t-tests there were differences between conditions. We found that both conditions and all array sizes were significantly above chance and above what would be expected by a "blue the red" strategy (4-item Arrays: Hypothetical mean = 50%, Center-embedded: 85%, $t(46) = 13.10$, $p <. 001$; Cross-serial: 94%, $t(45) = 39.79$, $p <. 001$; 6-item Arrays: Hypothetical mean = 17%, Center-embedded: 77%, $t(46) = 22.86$, $p <. 001$; Cross-serial: 90%, $t(46) = 68.68$, $p <. 001$; 8-item Arrays: Hypothetical mean = 4%, Center-embedded: 75 %, $t(44) = 20.25$, $p <. 001$; Cross-serial: 85 %, $t(45) = 46.99$, $p <. 001$). Additionally, we ran a mixed effects logistic regression using condition, array size, and their interaction as predictors of correct responses. The maximal model including a by subject random effects interaction term had a singular fit and the interaction term was removed from the random effects structure. We found that the best fitting model included fixed effects of condition and array size (compared to a model not including condition: $\chi^2(4) = 187.27$, $p < .001$), but the inclusion of an interaction term did not improve the model ($\chi^2(2) = 1.23$, $p = .54$; see Fig. S1 & Table S8).

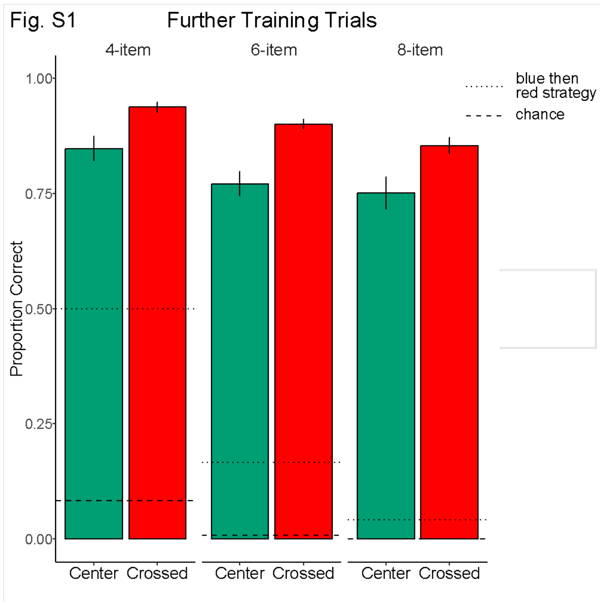

Figure S1: The proportion of correct sequences for each condition in the further training trials (with feedback Error bars represent the standard error of the mean. The dotted line represents predicted accuracy from a blue then red strategy (e.g., disregarding the base pairs) and the dashed line represents chance (random responding).

Table S8: Further training trials

	Trial Accuracy		
Predictors	*Odds Ratios*	*CI*	*p*
(Intercept)	10.26	6.70 – 15.69	**<0.001**
Condition [Crossed]	2.46	1.65 – 3.65	**<0.001**
Array Size [6Test]	0.40	0.28 – 0.56	**<0.001**
Array Size [8Test]	0.32	0.22 – 0.46	**<0.001**
**Random Effects**			
σ^2	3.29		
τ_{00} participant	1.81		
τ_{11} participant.ConditionCrossed	0.79		
τ_{11} participant.ArraySize6Test	1.20		
τ_{11} participant. ArraySize8Test	1.51		
ρ_{01}	-0.31		
	-0.65		
	-0.42		
ICC	0.27		
N participant	97		
Observations	9260		
Marginal R^2 / Conditional R^2	0.077 / 0.327		

Table S8: Fixed and Random effects from the logistic regression predicting trial accuracy from condition and array size.

Accuracy by position

To test if there were differences in accuracy for the second half of the 6- and 8-item lists, we ran a mixed effects logistic regression for both the 6- and 8-item array sizes using condition, touch number and their interaction to predict the accuracy of individual touches. The maximal model including by subject random effects of condition, touch number, and their interaction was used. In the 6-item length, the model had a singular fit and the interaction term was removed from the random effects structure. The best fitting model included main effects of condition and touch number for both the 6- and 8-item lengths compared to just a main effect of touch number (6-item: $\chi^2(1) = 17.18, p < .001$; 8-item: $\chi^2(4) = 10.48, p = .03$). The inclusion of the interaction term did not improve the model fit for either the 6- or 8- array length (6-item: $\chi^2(1) = 0.003, p = .96$; 8-item: $\chi^2(5) = 1.53, p = .91$; see Table S9). Accuracy was higher in the cross-serial condition and accuracy increased as the trial progressed, likely because the number of untouched items decreased.

Table S9: Accuracy by Item Number - Second half of list

	6-item Array			8-item Array		
Predictors	*Odds Ratios*	*CI*	*p*	*Odds Ratios*	*CI*	*p*
(Intercept)	0.54	0.20 – 1.41	0.207	1.98	0.80 – 4.90	0.138
Condition [Crossed]	2.70	1.73 – 4.22	**<0.001**	1.54	0.92 – 2.58	0.101
Touch	2.16	1.78 – 2.62	**<0.001**	1.55	1.34 – 1.79	**<0.001**
**Random Effects**						
σ^2	3.29			3.29		
τ_{00}	5.23 participant			5.12 participant		
τ_{11}	0.15 participant.Touch			0.56 participant.ConditionCrossed		
				0.13 participant.Touch		
ρ_{01}	-0.93 participant			-0.78		
				-0.83		
ICC	0.21			0.31		
N	94 participant			91 participant		
Observations	10090			12730		
Marginal R^2 / Conditional R^2	0.136 / 0.320			0.057 / 0.353		

Table S9: Fixed and Random effects from a logistic regression predicting Accuracy for individual touches using Condition and Touch Number (for the second half of the list) for both the 6- and 8-item array lengths.

Overall response time by condition

To test for overall response time differences between the conditions for second half of the lists, we ran a mixed effects linear regression using condition as a categorical predictor variable of overall

response time and subject as the random effects term for each array length (for each of the array lengths the maximal model with a by subject random effect of condition was singular or did not converge). Only correct responses were included in these response time analyses. We found that the best fitting model included a fixed effect of condition in the 6- and 8-item array lengths (6-item: $\chi^2(1) = 12.29$, $p < .01$; 8-item: $\chi^2(1) = 6.72$, $p < .01$; see Table S10 for complete model results). In the 4-item array length, the Null model was the best fitting model ($\chi^2(1) = 2.98$, $p = .08$).

Table S10: Overall Response Time - Second half of lists

	4-item List			6-item List			8-item List		
Predictors	*Estimates*	*CI*	*p*	*Estimates*	*CI*	*p*	*Estimates*	*CI*	*p*
(Intercept)	734.69	692.91 – 776.48	**<0.001**	892.09	827.23 – 956.96	**<0.001**	1024.17	945.41 – 1102.93	**<0.001**
Condition [Crossed]				-149.03	-234.29 – -63.77	**0.001**	-146.94	-257.32 – -36.55	**0.009**
**Random Effects**									
σ^2	66512.83			154016.78			306704.37		
τ_{00}	42795.88 participant			49495.42 participant			69145.17 participant		
τ_{11}				37181.32 participant.ConditionCrossed					
ρ_{01}				-0.59 participant					
ICC	0.39			0.21			0.18		
N	100 participant			94 participant			91 participant		
Observations	3296			9036			11180		
Marginal R^2 / Conditional R^2	0.000 / 0.392			0.027 / 0.236			0.014 / 0.195		

Table S10: Fixed and Random effects from linear regressions predicting total response time for the second half of the lists using Condition as the predictor variable for each array length.

Additionally, we ran a separate response time mixed effects linear regression using the same method as above on the first half of each list to make sure that there are no response time differences between the groups for the items that share the same order between the two conditions. We found that the best fitting model was the null model which did not include condition for each array length (4-item: $\chi^2(1) = 1.43$, $p = .23$; 6-item: $\chi^2(1) = 0.69$, $p = .41$; 8-item: $\chi^2(1) = 0.062$, $p = .80$; see Table S11 for complete model results).

Table S11: Overall Response Time - First half of lists

	4-item List			6-item List			8-item List		
Predictors	*Estimates*	*CI*	*p*	*Estimates*	*CI*	*p*	*Estimates*	*CI*	*p*
(Intercept)	1210.52	1098.97 – 1322.06	**<0.001**	1108.62	1021.25 – 1195.99	**<0.001**	1196.22	1039.09 – 1353.35	**<0.001**
**Random Effects**									
σ^2	1709781.61			5729958.89			39725520.54		
τ_{00}	262820.20 participant			123781.04 participant			248307.07 participant		
ICC	0.13			0.02			0.01		

N	100 participant	94 participant	91 participant
Observations	3296	9036	11180
Marginal R^2 / Conditional R^2	0.000 / 0.133	0.000 / 0.021	0.000 / 0.006

Table S11: Fixed and Random effects from linear regressions predicting total response time for the first half of the lists. The best fitting model did not include Condition as the predictor variable.

Main analyses (no exclusions)

To make sure that our exclusion criteria did not substantially affect our results, we reran the main analyses but included all subjects with no exclusions. First, we looked at the number of correct generalizations, which were qualitatively similar to the results with outliers removed (see Fig S2). We also ran the logistic regression predicting correct generalizations using condition (base case = center-embedded), array size (base case = 4-item), and their interaction. As with the main analysis, we found a significant difference between center-embedded and cross-serial trials (pairwise comparison collapsed across array lengths ($\beta_{condition}$ = -1.18, = .001).

Fig. S2 Phase 3: Novel Arrays (no outliers removed)

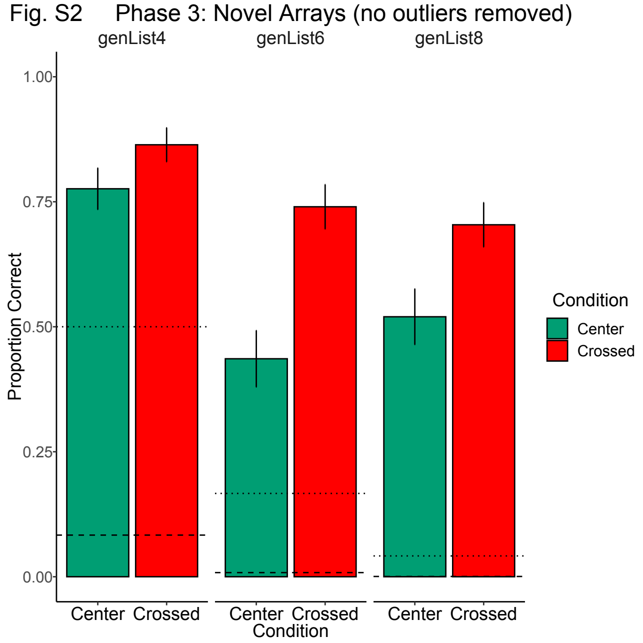

Figure S2. The proportion of fully correct sequences for each condition in Phase 3 for the test trials (no feedback). Error bars represent the standard error of the mean. The dotted line represents predicted accuracy from a blue then red strategy (e.g., disregarding the base pairs) and the dashed line represents chance (random responding).

Table S12: Phase 3 - Logistic Regression results. (No outliers excluded)

	Correct Generalizations		
Predictors	*Odds Ratios*	*CI*	*p*
(Intercept)	7.68	3.28 – 17.98	**<0.001**

Condition [Crossed]	2.43	0.68 – 8.76	0.174
Array Size [genList6]	0.07	0.03 – 0.17	**<0.001**
Array Size [genList8]	0.13	0.05 – 0.35	**<0.001**
Condition [Crossed] * Array Size [genList6]	4.55	1.12 – 18.54	**0.034**
Condition [Crossed] * Array Size [genList8]	1.44	0.34 – 6.18	0.623
**Random Effects**			
σ^2	3.29		
$\tau_{00\ participant}$	3.91		
$\tau_{11\ participant.ConditionCrossed}$	4.02		
$\tau_{11\ participant.ArraySizegenList6}$	3.25		
$\tau_{11\ participant.ArraySizegenList8}$	4.90		
ρ_{01}	-0.54		
	0.28		
	0.03		
ICC	0.62		
$N_{participant}$	100		
Observations	1500		
Marginal R^2 / Conditional R^2	0.145 / 0.676		

Table S12: Fixed and Random effects from the logistic regression predicting correct generalizations from condition, array size, and their interaction.

We also ran the response time analyses including all subjects (one subject in the center-embedded 8-item condition had no correct trials and thus could not be included). Again, we find qualitatively similar results, with the best fitting model including both main effects and the interaction term (6-item: $\chi^2(2) = 12.64$, $p = .002$, compared to the second-best fitting model with only Touch Number included; 8-item: $\chi^2(1) = 11.82$, $p < .001$, compared to the model not including the interaction; see Table S11 for complete model results).

Table S13: Response time by item number

	6-item Array			8-item Array		
Predictors	*Estimates*	*CI*	*p*	*Estimates*	*CI*	*p*
(Intercept)	1949.19	1626.92 – 2271.46	**<0.001**	2924.08	2381.68 – 3466.48	**<0.001**
Condition [Crossed]	-832.93	-1282.01 – -383.84	**<0.001**	-1394.27	-2146.18 – -642.37	**<0.001**
Touch	-206.26	-258.32 – -154.21	**<0.001**	-273.32	-341.96 – -204.67	**<0.001**

Condition [Crossed] * Touch	132.86	60.56 – 205.15	**<0.001**	169.81	74.83 – 264.78	**<0.001**
**Random Effects**						
σ^2	142972.02			402946.08		
τ_{00}	1013950.75 participant			3166294.79 participant		
τ_{11}	24277.84 participant.Touch			48915.80 participant.Touch		
ρ_{01}	-0.97 participant			-0.99 participant		
ICC	0.36			0.26		
N	100 participant			99 participant		
Observations	6404			8919		
Marginal R^2 / Conditional R^2	0.035 / 0.386			0.064 / 0.305		

Table S13: Fixed and Random effects from the linear regression predicting Response Time using Condition, Touch Number, and their interaction for both the 6- and 8-item array lengths. All outliers were included in this analysis. As with the other analyses predicting individual touch response times, we used the touch numbers from the second half of the list, not including the first touch in the second half of the list.

Response time analyses (80% accuracy exclusion criterion)

To make sure that the response time analyses hold when just including participants that aquired the correct grammar, we reran the response time analyses but included only subjects with >=80% accuracy on the further training trials for the given list length. We also ran the logistic regression predicting item-by-item response time using condition (base case = center-embedded), touch number (in the second half of the list), and their interaction. As with the main analysis, we found a significant interaction (and main effects) between condition and touch number such that the center-embedded condition had a steeper downward slope compared to the cross-serial condition for both array lengths (Table S14).

Table S14: Response time by item number (80% Accuracy Criterion)

	**6-item Array**			**8-item Array**		
Predictors	*Estimates*	*CI*	*p*	*Estimates*	*CI*	*p*
(Intercept)	1416.63	1193.54 – 1639.73	**<0.001**	1765.27	1468.39 – 2062.15	**<0.001**
Condition [Crossed]	-580.33	-864.40 – -296.26	**<0.001**	-612.40	-1000.08 – -224.73	**0.002**
Touch	-124.27	-162.68 – -85.86	**<0.001**	-140.22	-176.11 – -104.32	**<0.001**
Condition [Crossed] * Touch	90.60	41.69 – 139.51	**<0.001**	76.89	30.02 – 123.76	**0.001**
**Random Effects**						
σ^2	46819.98			85013.15		

τ_{00}	251835.78 $_{\text{participant}}$	487806.68 $_{\text{participant}}$
τ_{11}	7206.56 $_{\text{participant.Touch}}$	6791.92 $_{\text{participant.Touch}}$
ρ_{01}	-0.93 $_{\text{participant}}$	-0.97 $_{\text{participant}}$
ICC	0.45	0.35
N	68 $_{\text{participant}}$	58 $_{\text{participant}}$
Observations	4684	5974
Marginal R^2 / Conditional R^2	0.036 / 0.468	0.059 / 0.393

Text

Model

Setup and assumptions

A queue and stack memory architecture require different push operations (append item to list) and pop operations (remove item from list) in order to generate center-embedded and crossed- serial structures. In order to generate a center-embedded list with a stack, each blue item is chosen and pushed to memory; when generating red items, the matching blue items in memory are already in the correct order to generate a center-embedded structure when sequentially popped off, so they are simply read and the matching red item is chosen. Because the items are already in the correct order, this means that only a single operation is ever needed to generate each item (a push in the first half of the list to store the order the items were selected, and a pop in the second half of the list to retrieve the reverse order). To generate a center-embedded list with a queue, on the other hand, the blue items are in reverse order, so all items until the last remaining item in the queue must be sequentially popped off and stored in a second queue; the element matching the last item in the queue can then be accessed, read, and the matching red item can be chosen. So, in the 6-item condition for example, this entails a single operation for each item in the first half of the list (a push onto the queue). For the first item in the second half of the list, the first two items in the list need to be popped and stored in a new queue before popping the correct 3rd item at the back of the queue (for a total of 5 operations). This iterative popping and pushing onto a new queue makes essentially the same predictions that an iterative search with a pointer makes (longer processing time for items earlier in the second half of the sequence).

Conversely, in order to generate crossed-serial sequences using a stack, the blue items are initially in reverse order when sequentially popped off, so they must be stored (pushed) in a second stack in order to retrieve the first blue item that was chosen. Thus in the 6-item condition, for the first item in the second half of the list, you would need to pop, then push items on the top of the stack onto a new stack, then pop the last item and then make the correct match (a total of 5 operations, 3 pops and 2 pushes). However, once they have been pushed onto the second stack, the blue items are now in the correct order to be sequentially popped off, read, and matched. To generate crossed-serial structures using a queue simply requires sequentially popping off each blue item stored in memory and matching them (one operation, a pop, for each item). Tables S14-16 show the total number of push and pop operations required using a stack and queue in order to generate center-embedded (left) and crossed-serial (right) sequences across all sequence lengths tested.

Center-Embedded				
Touch number	1	2	3	4
Queue Ops	1	1	3	1
Stack Ops	1	1	1	1

Crossed-Serial				
Touch number	1	2	3	4
Queue Ops	1	1	1	1
Stack Ops	1	1	3	1

Table S14: Number of queue and stack operations for 4-item lists.

Center-Embedded						
Touch number	1	2	3	4	5	6
Queue Ops	1	1	1	5	3	1
Stack Ops	1	1	1	1	1	1

Crossed-Serial						
Touch number	1	2	3	4	5	6
Queue Ops	1	1	1	1	1	1
Stack Ops	1	1	1	5	1	1

Table S15: Number of queue and stack operations for 6-item lists.

Center-Embedded								
Touch number	1	2	3	4	5	6	7	8
Queue Ops	1	1	1	1	7	5	3	1
Stack Ops	1	1	1	1	1	1	1	1

Crossed-Serial								
Touch number	1	2	3	4	5	6	7	8
Queue	1	1	1	1	1	1	1	1
Stack	1	1	1	1	7	1	1	1

Table S16: Number of queue and stack operations for 8-item lists.

Response times

We assume that the time it takes to respond is dependent on the number of push/pop operations involved. For both structures, the reaction time is constant in the first half of the list under both a stack and queue, since there is no memory retrieval required. Stacks and queues therefore only make differing predictions about response times in the second half of the list, as shown in Tables 14-16. We included several response time parameters fit for each subject: an intercept representing the time required to make a choice (α_s); a start-up cost for the very first response, to capture the long latency of the very first choice (θ_s); a switch-cost, representing how long it takes to switch from choosing the first set of items (those in the first half of the list) to choosing matching items (γ_s), e.g., picking blue items to picking red items; and a slope, representing how long each push/pop operation takes (β_s).

The mean response time μ_s for a given subject s, given a function $P(\cdot)$ that takes as input the index and outputs the number of push/pop operations involved, is given by,

$$\mu_{s,i} = \alpha_s + \theta_s \cdot \mathbb{1}_{i=0} + \gamma_s \cdot \mathbb{1}_{S(i)} + \beta_s \cdot P(i), \tag{1}$$

where $\mathbb{1}$ is the indicator function. Here $\mathbb{1}_{i=0}$ is 1 only on the very first index and 0 otherwise; and $\mathbb{1}_{\mathrm{S(i)}}$ is 1 when switching between functions (represented by $S(i)$). The RT for a particular subject s on a particular index i is then drawn from,

$$RT_{s,i} \sim \text{LogNormal}(\mu_{s,i}, \sigma_s), \tag{2}$$

where σ_s is the standard deviation of response times.

Fig. S3 shows simulated response times to generate both structures using a queue (3a) and stack (3b) under a set of randomly sampled parameters. The black lines each represent one possible subject (i.e., particular parameters) and the predictions for each particular set of sampled parameters were made for both a queue and stack. The blue/orange lines show the average response times of these hypothetical subjects. The main difference in their predictions is in the center-embedded condition for 6- and 8-item lists, with the queue model having a high and downward trending RT slope in the second half of the list and the stack having a constant RT after the first item in the second half of the list.

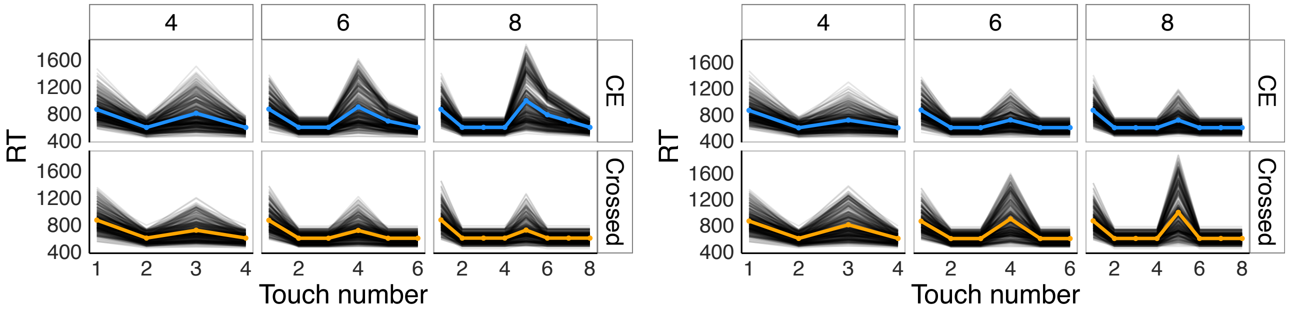

(a)

(b)

Fig. S3: Simulated reaction times under (a) the queue model and (b) the stack model. Each black line represents one possible subject (i.e., a particular set of parameters). The set of hypothetical subjects (parameters) shown here is the same in the queue and stack model — the only difference is whether they are assumed to be using a queue or stack to store items in memory.

Sources of error

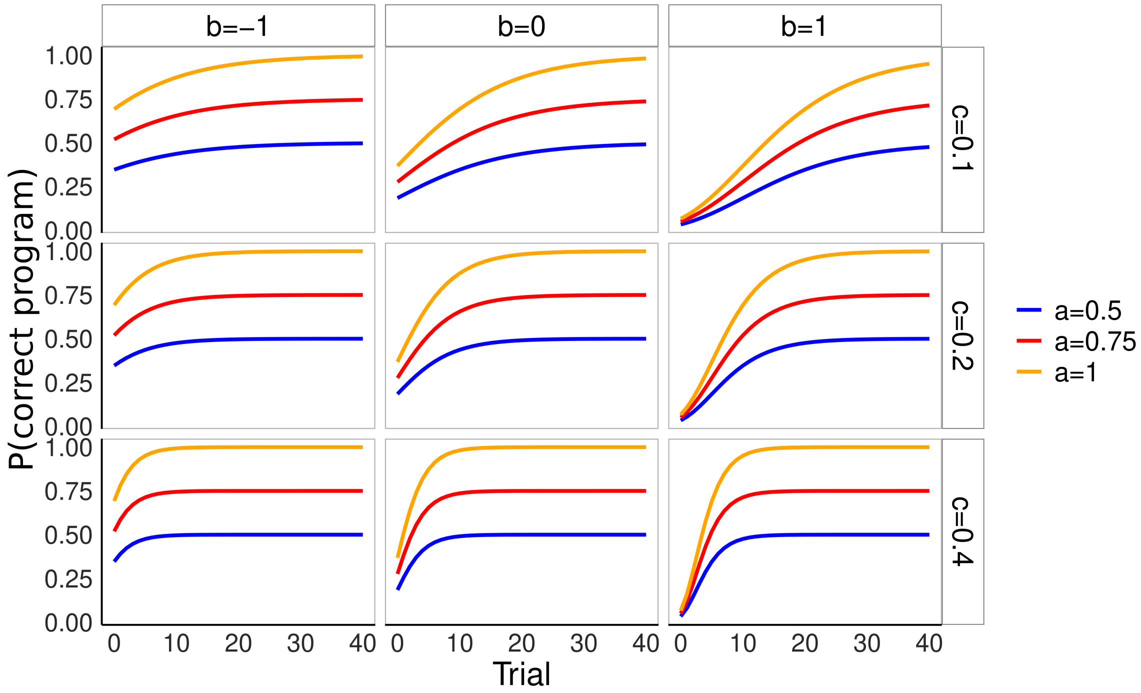

Fig. S4: The probability of correct program use over time under a Gompertz function, for different values of a, b, and c.

We assume that there are three potential reasons people make errors: 1) they are trying to generate the wrong structure or no structure at all beyond blue then red; 2) they forgot either which items they have already chosen or the order they chose them; or 3) they are not trying or paying attention. To account for the first of these possibilities, we assume that participants use the correct program on some proportion of trials, $G(t; a_s, b_s, c_s)$, where G is the Gompertz function and a_s, b_s, and c_s are subject parameters and t is the trial number. The Gompertz function is given by,

$$G(t; a_s, b_s, c_s) = a_s \cdot \mathrm{e}^{-\mathrm{e}^{b_s - c_s \cdot t}}. \quad (3)$$

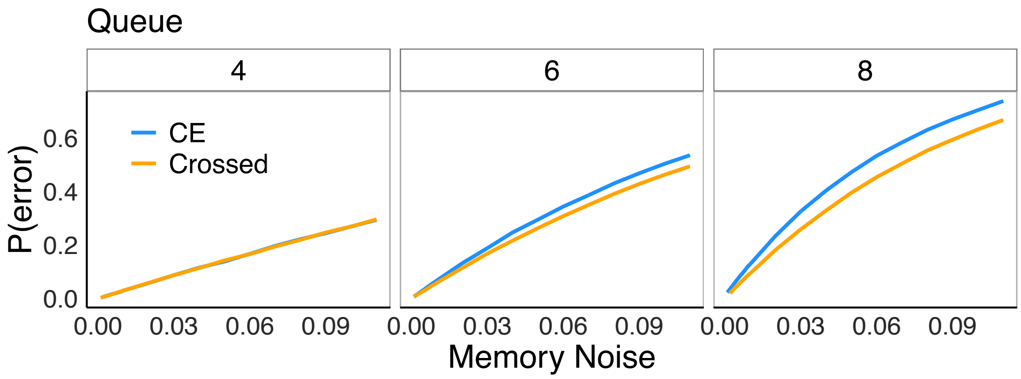

(a)

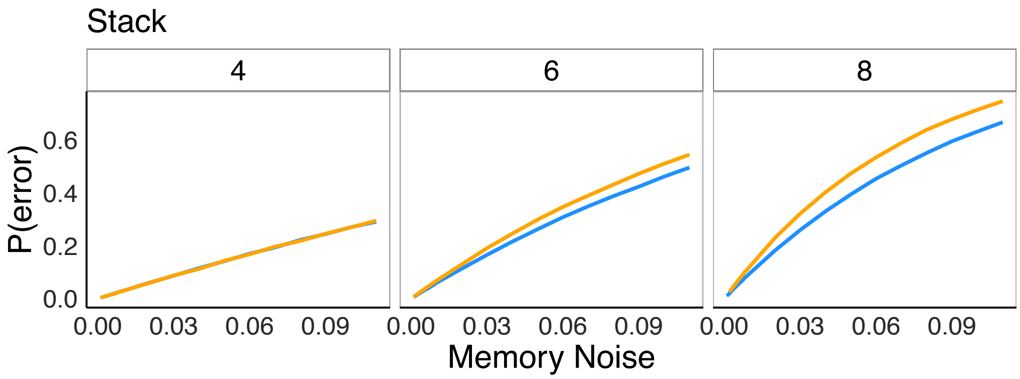

(b)

Fig. S5: The probability of an error in generating 4-, 6-, and 8-item lists as a function of the amount of memory noise, for both center-embedded structures (blue) and crossed-serial structures (orange), using (a) a queue and (b) a stack.

Here, a_s sets the asymptote, b_s sets the displacement on the x-axis; and c_s sets the slope (i.e., the learning rate). Fig. S4 illustrates the Gompertz function under different values of a_s, b_s, and c_s. On the trials in which participants were *not* using the correct program, we assumed they were generating sequences where the red items did not match the blue items in any systematic order (or in some consistent incorrect order). That is, they picked random red items instead of ones that matched blue items, where "matched" here means either in crossed-serial or center-embedded order depending on condition. Because this model is only fit to the training trials, in which participants were stopped as soon as they made an error, we could not differentiate between various possible incorrect programs beyond picking red items first and then non-matching blue items.

To account for mis-remembering which items were pressed or order, we assumed that each time an item was pushed into memory, there was some probability that memory is corrupted. For simplicity, we assumed that corruptions of items' order and deletions from memory were equally probable. Fig. 5 shows how the probability of making an error on a given trial scales with memory noise under both a queue (a) and stack (b), when generating both center-embedded and crossed-serial structures. The

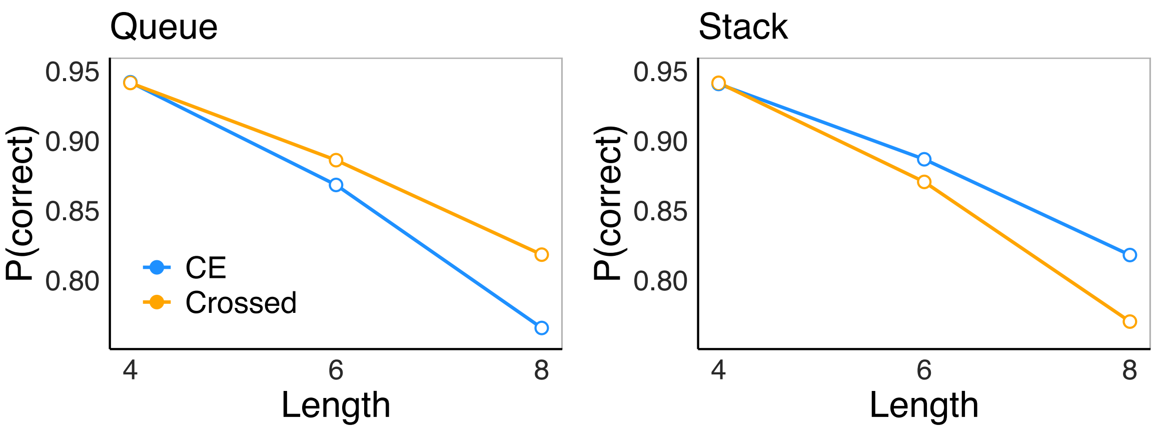

(a) (b)

Fig. S6: The probability of an error in generating 4-, 6-, and 8-item lists given memory noise of 0.02, for both center-embedded structures (blue) and crossed-serial structures (orange), under (a) a queue and (b) a stack.

two memory structures make differing predictions about the relative error rates for generating center-embedded and crossed-serial structures: the queue model predicts more errors for generating center-embedded sequences at longer lengths, whereas the stack model predicts more errors for generating crossed-serial sequences at longer lengths.

Fig. 6 shows the predictions for the rate of errors in both conditions under both a queue and stack given a fixed memory noise of 0.02. There is no difference in predicted error rates between queues and stacks, nor between center-embedded and crossed-serial structures, for 4-item sequences. However, for 6-item and 8-item sequences, queues and stacks make opposing predictions about how accurately center-embedded and crossed-serial structures can be generated. Using a queue, crossed-serial structures are generated with less error; using a stack, center-embedded structures are generated with less error. However, queues and stacks predict greater error rates for both types of sequences at longer lengths.

Model fitting

We fit each participant under both a queue and a stack across the entire training data. All parameters, except for the rate of correct program use, were assumed to be constant across the entire experiment. We found maximum-likelihood parameter estimates for each subject using a random search method: 1) randomly sample a set of parameters; 2) add normal noise to each parameter; 3) accept the new parameters if the likelihood of the new parameters is greater than the original; 4) repeat from step 2 until the average increase in log likelihood over 500 steps is less than 0.01. We verified that this fitting procedure could recover the parameters from simulated data, using randomly sampled parameters.

Inferred RT parameters

We show the inferred reaction time parameters in Fig.S 7. The inferred parameters in the queue model are shown in Fig. 7a and the inferred parameters for the stack model are shown in Fig. 7b.

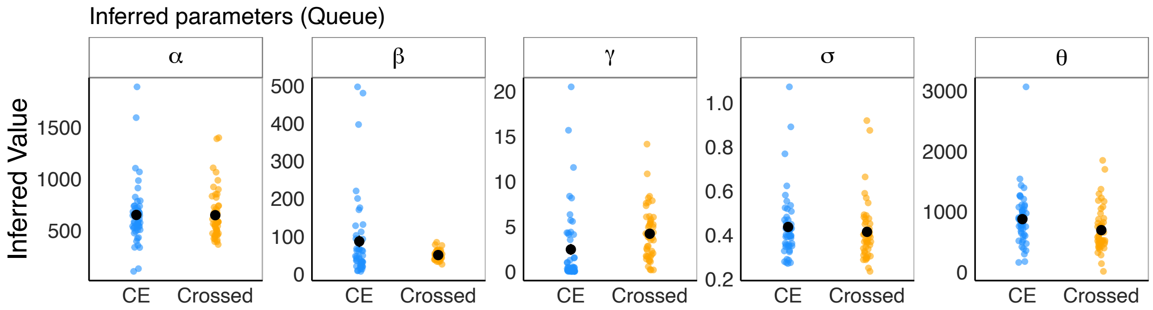

(a)

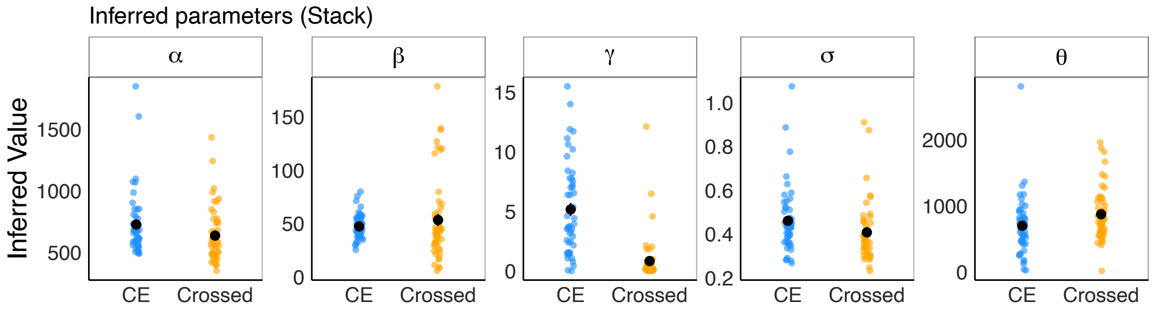

(b)

Fig. S7: The inferred parameters relating to response times across all subjects in the queue model (a) and stack model (b). Individual subjects' parameter values are shown in each panel as blue points for the center-embedded condition (left) and orange points in the crossed-serial condition (right). The black points indicate the mean inferred parameter value.

Both panels show the inferred RT intercept (α_s); the start-up RT cost (θ_s) for the very first response; the RT switch-cost (γ_s) for how long it takes to switch from one function to another; the RT per operation slope (β_s) for how long each operation takes; and the standard deviation of RTs (σ_s).

As expected, the inferred values of most parameters are quite similar for the stack and queue architectures, though there are two differences worth noting. The first is that the inferred β value in the queue model ($M = 66$, $CI = [58, 75]$) is higher than in the stack model ($M = 50$, $CI = [47, 53]$), which is consistent with the operations in the queue model capturing more of the variance in reaction times than the operations in the stack model. The second is that the stack model infers extremely different γ parameters between the two conditions — $M = 4.9$ ($CI = [4.4, 5.4]$) for center-embedded and $M = 1.5$ ($CI = [0.8, 2.4]$) for crossed-serial — even though these values should be identical (and would be had the experiment used within-subjects conditions). This is reflective of the fact that the stack model significantly underestimates reaction times on the first trial of the second half of the 6- and 8-item lists in the center-embedded condition but not the crossed-serial condition, so it infers a higher switch cost in the former.
